# Factors associated with an unfavorable outcome according to age in patients with COVID-19 admitted to intensive care in mainland France during the first three periods of the pandemic: a nationwide cohort study

**DOI:** 10.3389/fmed.2026.1816657

**Published:** 2026-04-23

**Authors:** Antoine Journé, Sabrina Tessier, Anna Maisa, Cécile Durand, Delphine Viriot, Isabelle Parent du Châtelet, Olivier Retel, Jean-Pierre Quenot, Christine Binquet

**Affiliations:** 1 Université Bourgogne Europe, CHU Dijon Bourgogne, Centre d’Investigation Clinique, Module épidémiologie clinique, INSERM, CIC1432, Dijon, France; 2 Santé publique France (French National Public Health Agency), Saint-Maurice, France; 3 Université Bourgogne Europe, CHU Dijon Bourgogne, Service de Médecine Intensive et Réanimation, INSERM, U1231, LIPNESS, LabEx LipSTIC, Dijon, France; 4 Société de Réanimation de Langue Française, Paris, France

**Keywords:** acute respiratory distress syndrome, COVID-19, France, intensive care unit, invasive respiratory support, mortality

## Abstract

**Background:**

Coronavirus disease 2019 (COVID-19) can affect multiple organs, especially the lungs, which may lead to intensive care unit (ICU) admission in the case of acute respiratory distress syndrome (ARDS). Other unfavorable outcomes can occur such as need for orotracheal intubation (OTI) and/or extracorporeal membrane oxygenation (ECMO) and even death. We took advantage of national surveillance data from ICU admissions managed by Santé publique France to investigate the factors associated with mortality, severe ARDS, ICU-free days as well as need for invasive ventilatory support in mainland France between February 2020 and June 2021.

**Methods:**

This nationwide cohort study analyzed critically ill COVID-19 patients admitted to ICU. We used Fine and Gray’s model and linear and logistic regressions to assess the factors associated with different outcomes. The main variable of interest was the first three periods of the pandemic: period 1 (February to July 2020), period 2 (August to December 2020) and period 3 (January to June 2021). We stratified all analyses according to predefined age groups: <45, 45–64 and ≥65 years.

**Results:**

The 15,423 included patients were mainly men (70%). Mean age was 64.1 ± 13.0 years. Mortality remained high throughout all three pandemic periods. The third pandemic period was associated with a higher risk of severe ARDS in patients aged ≥65 years as well as more ICU-free days and less use of invasive respiratory support regardless of age. Obesity was associated with a lower risk of death in patients aged ≥45 and a higher risk of severe ARDS and requiring invasive respiratory support in patients aged ≥65. Male sex was associated with a higher risk of death regardless of age, a higher risk of severe ARDS in patients aged ≥45 as well as fewer ICU-free days and a higher risk of using invasive respiratory support in patients aged ≥65.

**Conclusion:**

Prognosis did not significantly improve over time for COVID-19 patients admitted to ICU, however our findings highlight obesity and male sex as key factors in most severe COVID-19 cases, particularly in the elderly. This study also showed a reduction in the use of invasive respiratory support, irrespective of patient severity.

## Introduction

In December 2019, cases of pneumonia of unknown etiology were reported in Wuhan, China (1), marking the emergence of coronavirus disease (COVID-19). The causative agent, severe acute respiratory syndrome coronavirus 2 (SARS-CoV-2), was identified in February 2020 (2). The infection rapidly spread worldwide, causing millions of deaths, and was declared a public health emergency of international concern by the WHO in January 2020 (3). In France, the first imported cases were confirmed in January 2020 (4), followed by several clusters (5).

To limit transmission and prevent healthcare system overload, France implemented a strict nationwide lockdown on 16 March 2020, restricting movement to essential activities, including medical care, for 55 days (6). This measure was justified by asymptomatic transmission (7) and the risk of severe disease. Beyond respiratory involvement (8), SARS-CoV-2 infection could affect multiple organs, including the heart (9) and digestive system (10), and could lead to acute respiratory distress syndrome (ARDS) requiring intensive care unit (ICU) admission (11), at a time when ICU bed capacity was limited. Monitoring ICU admissions was therefore essential to adapt capacity to evolving needs.

In France, ICU surveillance was rapidly implemented using an existing influenza monitoring system. Nearly 200 ICUs nationwide reported data on patients admitted with COVID-19 to the French National Agency of Public Health [Santé publique France (SpF)] from the end of February 2020 (5). This national dataset provides a unique opportunity to examine temporal changes in ICU outcomes during the pandemic.

A key feature of the pandemic has been the occurrence of successive waves. During the first three periods (February 2020 to June 2021), ICU mortality may have decreased owing to increasing clinical experience in managing COVID-19–related ARDS and improved understanding of its pathophysiology. National surveillance data allow assessment of temporal variations in mortality across these periods. However, ICU admission criteria may also have evolved, potentially modifying the severity profile of admitted patients. Evaluating whether progressively more severe ARDS cases were admitted over time is therefore important.

ICU length of stay is another major outcome, reflecting both complication management (12) and associated societal costs (13). Changes in medical practices, particularly the use of invasive respiratory support such as orotracheal intubation (OTI) and extracorporeal membrane oxygenation (ECMO), likely occurred across successive periods and can be investigated using national data.

Because age strongly influences comorbidity distribution and COVID-19 prognosis (14), all analyses were stratified by age group. The primary objective of this study was to assess whether ICU mortality differed across the first three pandemic periods according to age. Secondary objectives were to evaluate, by age group, whether these periods were associated with severe ARDS, ICU-free days, and the need for invasive respiratory support.

## Materials and methods

### Data source

In France, an ICU surveillance system (adult and pediatric services) for severe influenza cases was set up in 2009 following the A(H1N1) influenza pandemic (15). This ongoing surveillance system relies on a coordinated effort by the regional offices of SpF and until 2018, it applied to all authorized ICUs. The initial objective of this surveillance was to collect clinical and virological data to assess the severity of influenza epidemics. The national surveillance evolved toward a sentinel system in 2018. At the end of February 2020, SpF used the existing network of ICUs to establish an epidemiological surveillance of severe COVID-19 cases with the aim to describe the characteristics and management of cases admitted to ICUs. Additional ICUs were recruited as sentinel sites during the COVID-19 pandemic, with a total of 186 ICUs, including 165 located in mainland France.

### Data collection

Physicians of the participating ICUs reported individual data on severe cases of COVID-19 using a one-page standardized notification form, which was forwarded to the regional offices of SpF. COVID-19 cases were confirmed by reverse transcription polymerase chain reaction. Cases of pediatric inflammatory multisystem syndrome (PIMS) were not included, as a specific surveillance system was put in place by SpF and French pediatric societies to monitor these atypical clinical cases (16). Each questionnaire was entered into a dedicated standardized web-based application (Voozanoo) (17) by epidemiologists from each regional office of SpF. The ICU surveillance system was authorized by the French data protection authority (Commission Nationale Informatique et Libertés; CNIL).

### Inclusion and exclusion criteria

We included all confirmed COVID-19 cases admitted to ICU in mainland France that were reported in the ICU surveillance system from February 2020 to June 2021. Patients were excluded from the analysis if they had another reason for hospitalization (e.g., COVID-19 diagnosis during their ICU stay when admitted for another reason), if their evolution was not known (unknown date of discharge and/or vital status) and if data on sex, age or major comorbidities were missing.

### Outcomes and variables of interest

The primary endpoint was defined as the death of the patient during ICU stay. The secondary endpoints included the occurrence of severe ARDS (P/F ratio ≤ 100) (18) and ICU-free days as the number of days during the first 30 days of hospitalization that the patient was alive and not admitted to ICU (19, 20). ICU-free days were equal to 0 if the patient dies before 30 days or if the patient was hospitalized in ICU for 30 days or more and ICU-free days were equal to 30-x if the patient was discharged from ICU before 30 days, considering x as the number of days spent in ICU. Invasive respiratory support during ICU stay were also considered as secondary outcomes.

The main variable of interest was the first three periods of the pandemic: (1) from 23 February to 31 July 2020; (2) from 1 August to 31 December 2020; and (3) from 1 January to 30 June 2021 (21). We also accounted for the geographic area and the number of cases declared by ICUs (<50, 50–99, ≥100 cases). As age has a differential effect on the distribution of comorbidities (14) and the association between age and the risk of developing a severe form of COVID-19 has been well documented since the start of the pandemic (22, 23), we stratified all analyses according to predefined age groups: <45, 45–64 and ≥65 years (24). Other variables considered in the analysis were the following: sex; body mass index (BMI) (categorized according to WHO guidelines: <18, 18–24, 25–29, 30–34, 35–39, ≥40 kg/m^2^) (25); and comorbidities including cardiac diseases, pulmonary diseases, kidney diseases, neuromuscular diseases, cancer, immunodeficiency, diabetes, and high blood pressure (HBP).

### Statistical analyses

Quantitative variables are presented as means and standard deviations (SD) for normally distributed variables and otherwise as median and interquartile range (IQR), while categorical variables are expressed as number of patients and percentages.

For each objective, patients with missing data for the outcome of interest were excluded. The percentage of missing data for each explanatory variable was described. For data missing completely at random, complete case analyses were performed. If data were missing conditionally at random and with a missing rate <15%, a multiple imputation method was applied. In other cases, a missing data category was created for the concerned variable.

For the main analysis assessing the factors associated with mortality, baseline was defined as ICU admission, and the delay for survival analysis was calculated as the time from ICU admission to death during the ICU stay for deceased patients and from ICU admission to discharge otherwise. However, the censorship of observations for surviving patients based on ICU discharge could not be considered independent from the outcome. Thus, to estimate the association between potential prognostic factors and mortality during the ICU stay, we used Fine and Gray’s model (26). Variables associated with the risk of death during the ICU stay with a *p* < 0.2 in univariable analyses were selected for inclusion in multivariable analyses. Backward stepwise was applied to select variables independently associated with the risk of death based on the likelihood ratio test at an alpha risk of 0.05. Variables deemed epidemiologically relevant (forced variables) were retained, regardless of their statistical significance. Interactions between pandemic periods and other variables retained in the final model were systematically checked (alpha risk defined according to the Bonferroni correction). If one of the tested interactions appeared significant, the analysis was stratified accordingly. Model adequacy was assessed using H-H plots. Results were expressed as subdistribution hazard ratio (SHR) with 95% confidence interval (CI).

To investigate associations between the covariates and ICU-free days, linear regression models were used. A selection procedure similar to that used for the main analysis was applied. The quality of the linear models was assessed by representing the residuals using histograms and specifying the coefficient of determination (R2) adjusted for each model. Associations were expressed as coefficients of the linear regressions and their 95% CI.

Associations between covariates and the use of invasive ventilatory support in ICU as well as severe ARDS occurrence were estimated using logistic regression models. The same variable selection strategy was applied to the other analyses. The quality of the logistic regression was assessed by estimating the C-statistic and 95% CI and performing Hosmer-Lemeshow tests. Associations were expressed as odds ratio (OR) and 95% CI.

For each objective, pandemic periods, pandemic pressure, geographic location of the ICU and patient sex were forced.

All statistical analyses were carried out using R Studio software version 2024.04.0.

## Results

### Population study

**Figure 1:**
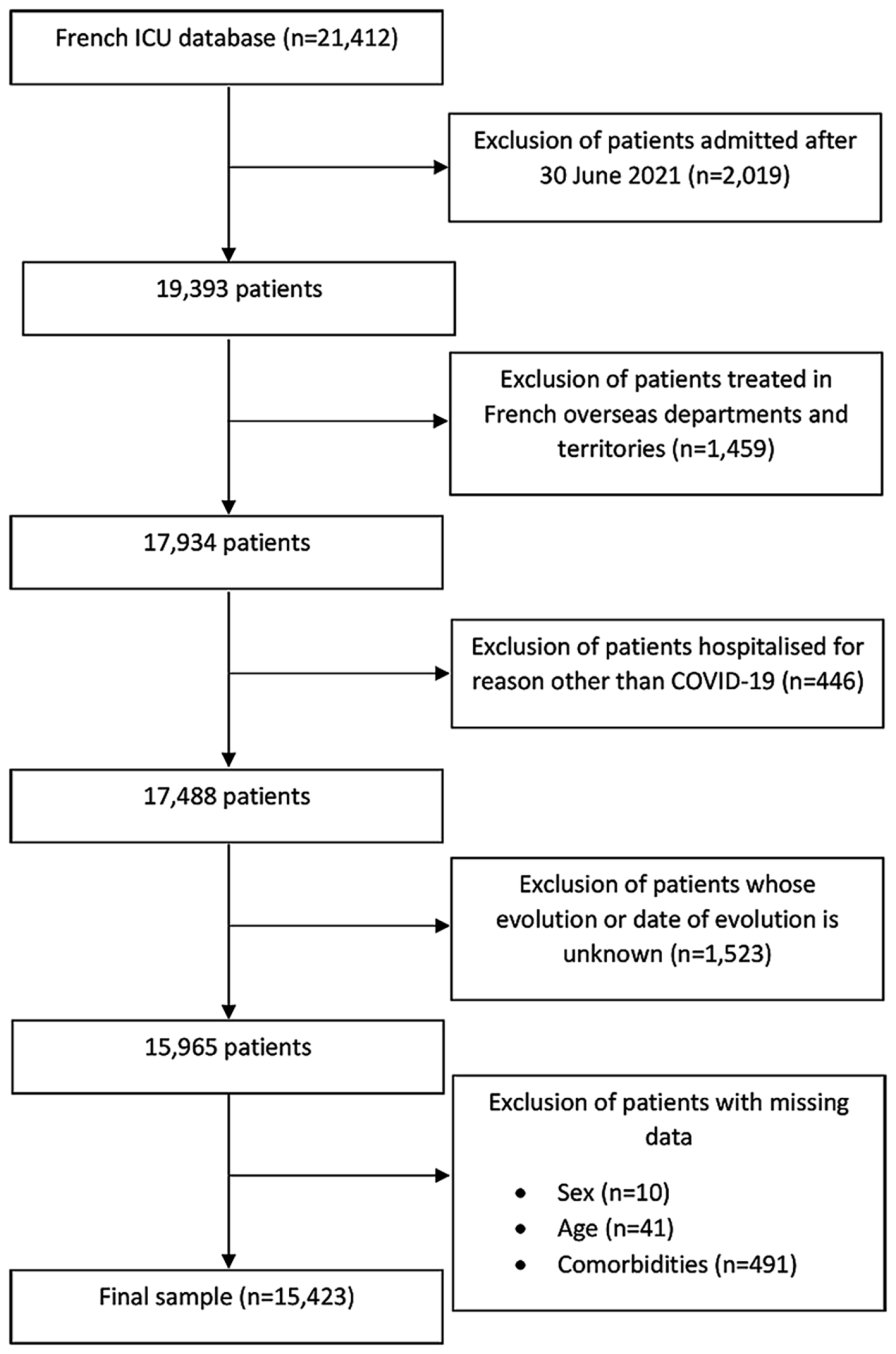
Selection of patients with COVID-19 admitted to intensive care in mainland France, February 2020–June 2021.

From February 2020 to June 2021, 21,412 patients were recorded in the ICU surveillance system. We excluded patients admitted after 30 June 2021 (*n* = 2,019), those treated in French overseas departments and territories (*n* = 1,459), and those hospitalized for reasons other than COVID-19 (*n* = 446). Data on their vital status and/or length of stay were missing for 1,523 patients, while for 542 additional patients, data on sex, age and/or major comorbidities were missing, resulting in 15,423 (88.2%) critically ill patients with laboratory-confirmed COVID-19 for our analyses (Figure 1). Briefly, excluded patients were younger, with a lower BMI and fewer comorbidities; they less often suffered from severe ARDS (see Supplementary File 1).

The 15,423 included patients were mainly men (70%) (Table 1). Mean age was 64.1 ± 13.0 years overall, 63.6 ± 13.5 years from 23 February to 31 July 2020, 66.3 ± 12.4 years from 1 August to 31 December 2020 and 63.0 ± 12.9 years from 1 January to 30 June 2021. The sex ratio (men/women) was lower in patients <45 years (1.58 vs. 2.44 and 2.32, respectively, for patients between 45 and 64 years and for those ≥65 years). Older patients had more cardiovascular comorbidities and the frequency of severe ARDS increased with age (27% in patients<45 years vs. 48% in those aged ≥65 years), as well as use of invasive ventilatory support (36% in patients<45 years vs. 53% in those aged ≥65 years) and death (from 5% in patients <45 years to 12% between 45 and 64 years and 31% in those aged ≥65 years). Conversely, younger patients more often had a BMI ≥ 30 kg/m^2^ (54% for patients <45 years vs. 34.4% for patients ≥65 years).

**Table 1:** Description of patients with COVID-19 admitted to intensive care by age group.

Variables	Overall (*n* = 15,423)^1^	<45 years (*n* = 1,137)^1^	45–64 years (*n* = 5,406)^1^	≥65 years (*n* = 8,880)^1^
Sex				
Female	4,684 (30)	441 (39)	1,572 (29)	2,671 (30)
Male	10,739 (70)	696 (61)	3,834 (71)	6,209 (70)
Number of reports per ICU				
<50	820 (5.3)	116 (10)	254 (4.7)	450 (5.1)
50–99	1,278 (8.3)	81 (7.1)	441 (8.2)	756 (8.5)
≥100	13,325 (86)	940 (83)	4,711 (87)	7,674 (86)
Pandemic periods (ICU admission date)				
23 February to 31 July 2020	3,267 (21)	263 (23)	1,177 (22)	1,827 (21)
1 August to 31 December 2020	4,860 (32)	267 (23)	1,453 (27)	3,140 (35)
1 January to 30 June 2021	7,296 (47)	607 (53)	2,776 (51)	3,913 (44)
Region of care				
Île-de-France	735 (4.8)	87 (7.7)	295 (5.5)	353 (4.0)
Auvergne-Rhône-Alpes	1,829 (12)	130 (11)	613 (11)	1,086 (12)
Bourgogne-Franche-Comté	1,193 (7.7)	73 (6.4)	318 (5.9)	802 (9.0)
Bretagne	583 (3.8)	51 (4.5)	195 (3.6)	337 (3.8)
Corse	126 (0.8)	8 (0.7)	38 (0.7)	80 (0.9)
Centre-Val de Loire	619 (4.0)	47 (4.1)	242 (4.5)	330 (3.7)
Grand Est	251 (1.6)	12 (1.1)	96 (1.8)	143 (1.6)
Hauts-de-france	1,975 (13)	143 (13)	735 (14)	1,097 (12)
Nouvelle-aquitaine	1,273 (8.3)	86 (7.6)	460 (8.5)	727 (8.2)
Normandie	977 (6.3)	52 (4.6)	354 (6.5)	571 (6.4)
Occitanie	2,244 (15)	161 (14)	732 (14)	1,351 (15)
Provence-Alpes-Côte d’Azur	2,014 (13)	153 (13)	744 (14)	1,117 (13)
Pays de la Loire	1,604 (10)	134 (12)	584 (11)	886 (10)
Maximum invasive ventilatory support used during stay				
Neither OTI nor ECMO	7,044 (46)	642 (56)	2,638 (49)	3,764 (42)
OTI and/or ECMO	7,563 (49)	407 (36)	2,473 (46)	4,683 (53)
Missing data	816 (5.3)	88 (7.7)	295 (5.5)	433 (4.9)
Maximum ARDS reached during stay				
Absence	2,123 (14)	307 (27)	802 (15)	1,014 (11)
Mild	1,053 (6.8)	109 (9.6)	439 (8.1)	505 (5.7)
Moderate	4,124 (27)	316 (28)	1,528 (28)	2,280 (26)
Severe	6,734 (44)	312 (27)	2,124 (39)	4,298 (48)
Missing data	1,389 (9.0)	93 (8.2)	513 (9.5)	783 (8.8)
BMI by class (in kg/m^2^)				
<18	48 (0.3)	7 (0.6)	12 (0.2)	29 (0.3)
18–24	1,955 (13)	102 (9.0)	520 (9.6)	1,333 (15)
25–29	4,316 (28)	197 (17)	1,441 (27)	2,678 (30)
30–34	3,519 (23)	263 (23)	1,352 (25)	1,904 (21)
35–39	1,635 (11)	158 (14)	670 (12)	807 (9.1)
≥40	1,041 (6.7)	191 (17)	467 (8.6)	383 (4.3)
Missing data	2,909 (19)	219 (19)	944 (17)	1,746 (20)
Comorbidities				
Cardiac diseases	3,329 (22)	64 (5.6)	650 (12)	2,615 (29)
Pulmonary diseases	3,072 (20)	152 (13)	946 (17)	1,974 (22)
Renal diseases	1,134 (7.4)	52 (4.6)	260 (4.8)	822 (9.3)
Hepatic diseases	289 (1.9)	13 (1.1)	118 (2.2)	158 (1.8)
Neuromuscular diseases	477 (3.1)	30 (2.6)	134 (2.5)	313 (3.5)
Cancer	787 (5.1)	21 (1.8)	206 (3.8)	560 (6.3)
Immunodeficiency	1,027 (6.7)	70 (6.2)	371 (6.9)	586 (6.6)
Diabetes (types 1 and 2)	4,317 (28)	137 (12)	1,319 (24)	2,861 (32)
High blood pressure	6,491 (42)	142 (12)	1,750 (32)	4,599 (52)
Other comorbidities	2,043 (13)	189 (17)	713 (13)	1,141 (13)
Vital status at ICU discharge				
Death	3,451 (22)	58 (5.1)	638 (12)	2,755 (31)
Transfer out of or to another ICU, or hospital discharge	11,972 (78)	1,079 (95)	4,768 (88)	6,125 (69)
Length of stay in ICU (in days)	10 (5, 21)	6 (3, 13)	9 (4, 20)	11 (5, 22)

^1^n (%), Median (IQR). ARDS, acute respiratory distress syndrome, BMI, body mass index, ECMO, extracorporeal membrane oxygenation, ICU, intensive care unit, OTI, orotracheal intubation. A patient may have several comorbidities.

The sex ratio (men/women) decreased during the third pandemic period (1.9 for January to June 2021 vs. at least 2.6 for the previous periods, Table 2). Older patients were more often recorded from August to December 2020, with comorbidities being more frequent for patients recorded during this period. Conversely, severe ARDS remained stable during the three periods, although the use of invasive respiratory support decreased over time (from 65% during the first period to 45% during the third period).

**Table 2:** Description of patients with COVID-19 admitted to intensive care by pandemic period.

Variables	Overall (*n* = 15,423)^1^	23 February to 31 July 2020 (*n* = 3,267)^1^	1 August to 31 December 2020 (*n* = 4,860)^1^	1 January to 30 June 2021 (*n* = 7,296)^1^
Sex				
Female	4,684 (30)	901 (28)	1,351 (28)	2,432 (33)
Male	10,739 (70)	2,366 (72)	3,509 (72)	4,864 (67)
Number of reports per ICU				
<50	820 (5.3)	477 (15)	198 (4.1)	145 (2.0)
50–99	1,278 (8.3)	487 (15)	401 (8.3)	390 (5.3)
≥100	13,325 (86)	2,303 (70)	4,261 (88)	6,761 (93)
Age group (in years)				
<45	1,137 (7.4)	263 (8.1)	267 (5.5)	607 (8.3)
45–64	5,406 (35)	1,177 (36)	1,453 (30)	2,776 (38)
≥65	8,880 (58)	1,827 (56)	3,140 (65)	3,913 (54)
Region of care				
IDF	735 (4.8)	162 (5.0)	246 (5.1)	327 (4.5)
ARA	1,829 (12)	318 (9.7)	848 (17)	663 (9.1)
BFC	1,193 (7.7)	319 (9.8)	396 (8.1)	478 (6.6)
BRE	583 (3.8)	112 (3.4)	150 (3.1)	321 (4.4)
COR	126 (0.8)	34 (1.0)	27 (0.6)	65 (0.9)
CVL	619 (4.0)	178 (5.4)	102 (2.1)	339 (4.6)
GES	251 (1.6)	136 (4.2)	72 (1.5)	43 (0.6)
HDF	1,975 (13)	451 (14)	653 (13)	871 (12)
NAQ	1,273 (8.3)	294 (9.0)	312 (6.4)	667 (9.1)
NOR	977 (6.3)	234 (7.2)	288 (5.9)	455 (6.2)
OCC	2,244 (15)	401 (12)	721 (15)	1,122 (15)
PACA	2,014 (13)	373 (11)	525 (11)	1,116 (15)
PDL	1,604 (10)	255 (7.8)	520 (11)	829 (11)
Maximum invasive ventilatory support used during stay				
Neither OTI nor ECMO	7,044 (46)	889 (27)	2,271 (47)	3,884 (53)
OTI and/or ECMO	7,563 (49)	2,127 (65)	2,132 (44)	3,304 (45)
Missing data	816 (5.3)	251 (7.7)	457 (9.4)	108 (1.5)
Maximum ARDS reached during stay				
Absence	2,123 (14)	639 (20)	621 (13)	863 (12)
Mild	1,053 (6.8)	221 (6.8)	337 (6.9)	495 (6.8)
Moderate	4,124 (27)	912 (28)	1,253 (26)	1,959 (27)
Severe	6,734 (44)	1,384 (42)	2,060 (42)	3,290 (45)
Missing data	1,389 (9.0)	111 (3.4)	589 (12)	689 (9.4)
BMI by class (in kg/m^2^)				
<18	48 (0.3)	4 (0.1)	19 (0.4)	25 (0.3)
18–24	1,955 (13)	222 (6.8)	702 (14)	1,031 (14)
25–29	4,316 (28)	461 (14)	1,555 (32)	2,300 (32)
30–34	3,519 (23)	457 (14)	1,146 (24)	1,916 (26)
35–39	1,635 (11)	211 (6.5)	513 (11)	911 (12)
≥40	1,041 (6.7)	190 (5.8)	310 (6.4)	541 (7.4)
Missing data	2,909 (19)	1,722 (53)	615 (13)	572 (7.8)
Comorbidities				
Cardiac diseases	3,329 (22)	675 (21)	1,216 (25)	1,438 (20)
Pulmonary diseases	3,072 (20)	577 (18)	1,052 (22)	1,443 (20)
Renal diseases	1,134 (7.4)	210 (6.4)	414 (8.5)	510 (7.0)
Hepatic diseases	289 (1.9)	32 (1.0)	115 (2.4)	142 (1.9)
Neuromuscular diseases	477 (3.1)	127 (3.9)	160 (3.3)	190 (2.6)
Cancer	787 (5.1)	–	330 (6.8)	457 (6.3)
Immunodeficiency	1,027 (6.7)	247 (7.6)	379 (7.8)	401 (5.5)
Diabetes (types 1 and 2)	4,317 (28)	858 (26)	1,572 (32)	1,887 (26)
High blood pressure	6,491 (42)	1,001 (31)	2,232 (46)	3,258 (45)
Other comorbidities	2,043 (13)	371 (11)	724 (15)	948 (13)
Evolution				
Death	3,451 (22)	722 (22)	1,235 (25)	1,494 (20)
Transfer out of or to another ICU, or hospital discharge	11,972 (78)	2,545 (78)	3,625 (75)	5,802 (80)
Length of ICU (in days)	10 (5, 21)	13 (5, 25)	10 (5, 20)	9 (5, 19)

^1^n (%), Median (IQR). ARA, Auvergne-Rhône-Alpes, ARDS, acute respiratory distress syndrome, BFC, Bourgogne-Franche-Comté, BMI, body mass index, BRE, Bretagne, COR, Corse, CVL, Centre-Val de Loire, ECMO, extracorporeal membrane oxygenation, GES, Grand Est, HDF, Hauts-de-France, ICU, intensive care unit, IDF, Île-de-France, NAQ, Nouvelle-Aquitaine, NOR, Normandie, OCC, Occitanie, OTI, orotracheal intubation, PACA, Provence-Alpes-Côte d’Azur, PDL, Pays de la Loire. A patient may have several comorbidities. -: data not collected in period 1.

### Factors associated with ICU mortality

Univariate analyses are presented in Supplementary File 2. In multivariate analyses, regardless of patient age and after adjusting for the other covariates, mortality was similar for the three pandemic waves (Table 3). However, in younger patients (<45 years), as the number of events is very small (*n* = 58), we lack statistical power which could explain the non-significant result in this age group. In other age categories, the use of invasive respiratory support and severe ARDS were associated with mortality (*p* < 0.001). All comorbidities were associated with a higher risk of death except for HBP. Being overweight and obese were associated with a lower risk of death (even morbid obesity in older patients), while women also had a lower risk of death in the oldest age category. H-H plots are presented in Supplementary File 3.

**Table 3:** Factors associated with mortality by age group (*n* = 15,423), multivariate analysis.

Variables	<45 years (*n* = 1,137)		45–64 years (*n* = 5,406)		≥65 years (*n* = 8,880)	
	*SHR* (95% CI)^1^	*p*-value^1^	SHR (95% CI)^1^	*p*-value^1^	SHR (95% CI)^1^	*p*-value^1^
Sex						
Male	Ref	Ref	Ref	Ref	Ref	Ref
Female	0.94 (0.53–1.65)	0.83	0.97 (0.80–1.16)	0.71	0.89 (0.82–0.97)	0.01
Pandemic periods (ICU admission date)						
23 February to 31 July 2020	Ref	Ref	Ref	Ref	Ref	Ref
1 August to 31 December 2020	1.44 (0.77–2.72)	0.26	1.25 (0.98–1.59)	0.08	1.06 (0.95–1.19)	0.32
1 January to 30 June 2021	0.52 (0.25–1.06)	0.07	0.96 (0.76–1.22)	0.75	0.99 (0.88–1.11)	0.79
Maximum invasive ventilatory support used during stay						
Neither OTI nor ECMO	Ref	Ref	Ref	Ref	Ref	Ref
OTI and/or ECMO	5.96 (1.96–18.07)	0.002	3.76 (2.78–5.09)	<0.001	1.39 (1.25–1.55)	<0.001
Missing data	0.77 (0.19–3.19)	0.72	1.88 (1.12–3.15)	0.02	1.01 (0.80–1.27)	0.95
Maximum ARDS reached during stay						
Absence	Ref	Ref	Ref	Ref	Ref	Ref
Minor	0.77 (0.15–4.02)	0.76	0.61 (0.29–1.28)	0.19	0.92 (0.66–1.27)	0.60
Moderate	0.31 (0.07–1.30)	0.11	0.81 (0.49–1.33)	0.40	1.09 (0.87–1.37)	0.44
Severe	1.70 (0.48–6.08)	0.41	2.97 (1.83–4.80)	<0.001	4.25 (3.42–5.29)	<0.001
Missing data	2.01 (0.59–6.81)	0.26	2.08 (1.25–3.44)	0.005	2.75 (2.15–3.52)	<0.001
BMI by class (in kg/m^2^)						
<18	NR	NR	2.32 (0.98–5.49)	0.06	1.70 (0.87–3.27)	0.11
18–24	Ref	Ref	Ref	Ref	Ref	Ref
25–29	NR	NR	0.65 (0.50–0.84)	<0.001	0.83 (0.74–0.93)	0.001
30–34	NR	NR	0.48 (0.37–0.63)	<0.001	0.77 (0.68–0.88)	<0.001
35–39	NR	NR	0.63 (0.47–0.86)	0.003	0.71 (0.60–0.83)	<0.001
≥40	NR	NR	0.77 (0.55–1.07)	0.12	0.75 (0.60–0.93)	0.009
Missing data	NR	NR	0.80 (0.61–1.06)	0.13	1.07 (0.94–1.22)	0.33
Comorbidities						
Cardiac diseases	NR	NR	2.04 (1.68–2.47)	<0.001	1.36 (1.25–1.47)	<0.001
Pulmonary diseases	NR	NR	1.49 (1.23–1.80)	<0.001	1.33 (1.22–1.45)	<0.001
Renal diseases	NR	NR	1.62 (1.21–2.17)	0.001	1.62 (1.44–1.82)	<0.001
Hepatic diseases	NR	NR	2.35 (1.61–3.42)	<0.001	1.64 (1.30–2.07)	<0.001
Neuromuscular diseases	NR	NR	NR	NR	1.57 (1.30–1.89)	<0.001
Cancer	4.79 (1.61–14.61)	0.005	1.85 (1.38–2.49)	<0.001	1.56 (1.36–1.79)	<0.001
Immunodeficiency	NR	NR	2.04 (1.60–2.60)	<0.001	1.29 (1.13–1.48)	<0.001
Diabetes (types 1 and 2)	NR	NR	NR	NR	1.10 (1.01–1.19)	0.03
High blood pressure	2.53 (1.49–4.29)	<0.001	NR	NR	NR	NR
Other comorbidities	–	–	NR	NR	1.17 (1.06–1.30)	0.003

^1^Fine and Gray’s competitive risk model. ARDS, acute respiratory distress syndrome, BMI, body mass index, ECMO, extracorporeal membrane oxygenation, ICU, intensive care unit, OTI, orotracheal intubation, Ref, reference class, SHR, subdistribution hazard ratio, 95% CI: 95% confidence interval. Models adjusted to the number of reports per ICU. A patient may have several comorbidities. NR: not retained by the step-by-step algorithm. -: not significant in univariate analysis.

### Factors associated with severe ARDS

**Figure 2:**
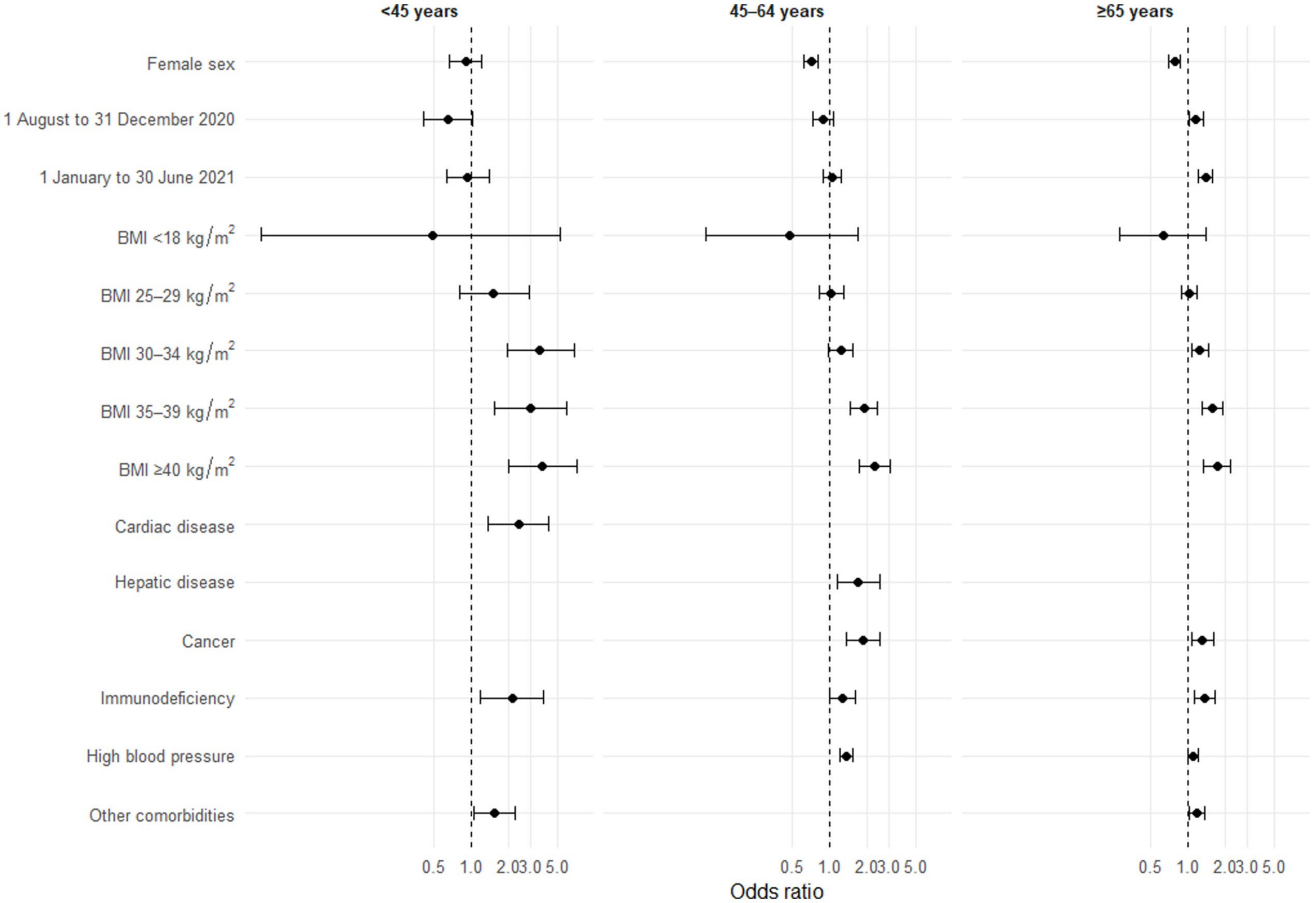
Forest plot of factors associated with severe versus absence/minor/moderate ARDS by age group (*n* = 14,034), multivariate analyses. ARDS, acute respiratory distress syndrome; BMI, body mass index. Models adjusted for number of reports per ICU and region of care. A patient may have several comorbidities. Some comorbidities are not presented, either because they were not significant in univariate analyses, or because they were not retained by the step-by-step algorithm. Missing categories are also not presented.

Overall, 14,034 patients admitted to ICU were included in this analysis, 48% of whom developed severe ARDS (see Supplementary File 4). Univariate analyses are presented in Supplementary File 5. Multivariate analysis showed a higher risk of severe ARDS after 1 August 2020 in patients aged ≥65 years, a lower risk of severe ARDS in women aged ≥45 compared to men of the same age category and a higher risk of severe ARDS in patients with a BMI ≥ 35 kg/m^2^ in all age categories compared to a BMI between 18 and 24 kg/m^2^ (Figure 2). Conversely, while immunodepression was associated with a higher risk of severe ARDS in all age categories, cardiac comorbidities were only associated with an increased risk of severe ARDS in patients <45 years. An increased risk was observed for patients aged ≥45 years with cancer or HBP. In our sample, hepatic diseases were only associated with severe ARDS in patients aged between 45 and 64 years. The quality of the multivariate models is presented in Supplementary File 6.

### Factors associated with ICU-free days

**Figure 3:**
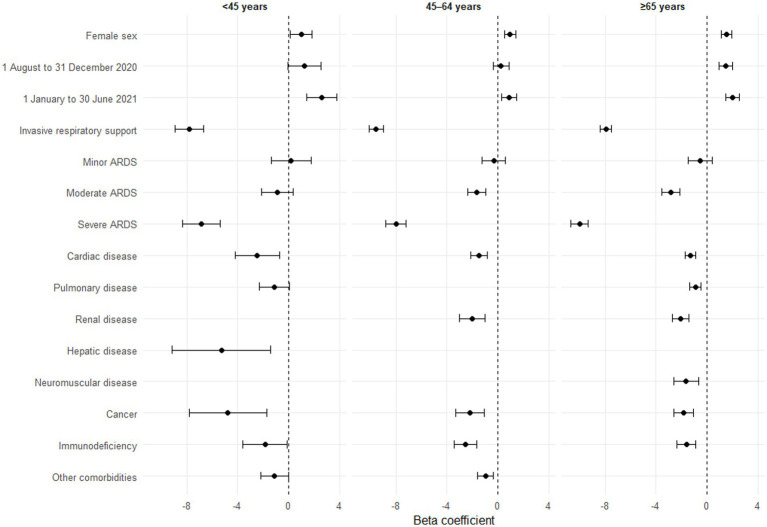
Forest plot of factors associated with ICU-free days by age group (*n* = 15,423), multivariate analyses. ARDS, acute respiratory distress syndrome, ECMO, extracorporeal membrane oxygenation, ICU, intensive care unit, OTI, orotracheal intubation. Models adjusted for number of reports per ICU and region of care. A patient may have several comorbidities. Some comorbidities are not presented, either because they were not significant in univariate analyses, or because they were not retained by the step-by-step algorithm. Missing categories are also not presented.

Univariate analyses are presented in Supplementary File 7. In multivariate analyses, ICU-free days increased over time (Figure 3). Women had more ICU-free days than men. Conversely, most comorbidities (cardiac disease, cancer, immunodeficiency) were associated with fewer ICU-free days independent of patient age. Patients aged <45 years with hepatic disease, those aged ≥45 with renal disease and those aged ≥65 with neuromuscular disease or a previous history of pulmonary disease also had fewer ICU-free days. Of note, only morbid obesity in patients <45 years was associated with fewer ICU-free days. Severe ARDS and the use of invasive respiratory support were associated with fewer ICU-free days regardless of age compared to patients without these conditions. Histograms of residuals are presented in Supplementary File 8.

### Factors associated with the use of invasive respiratory support

**Figure 4:**
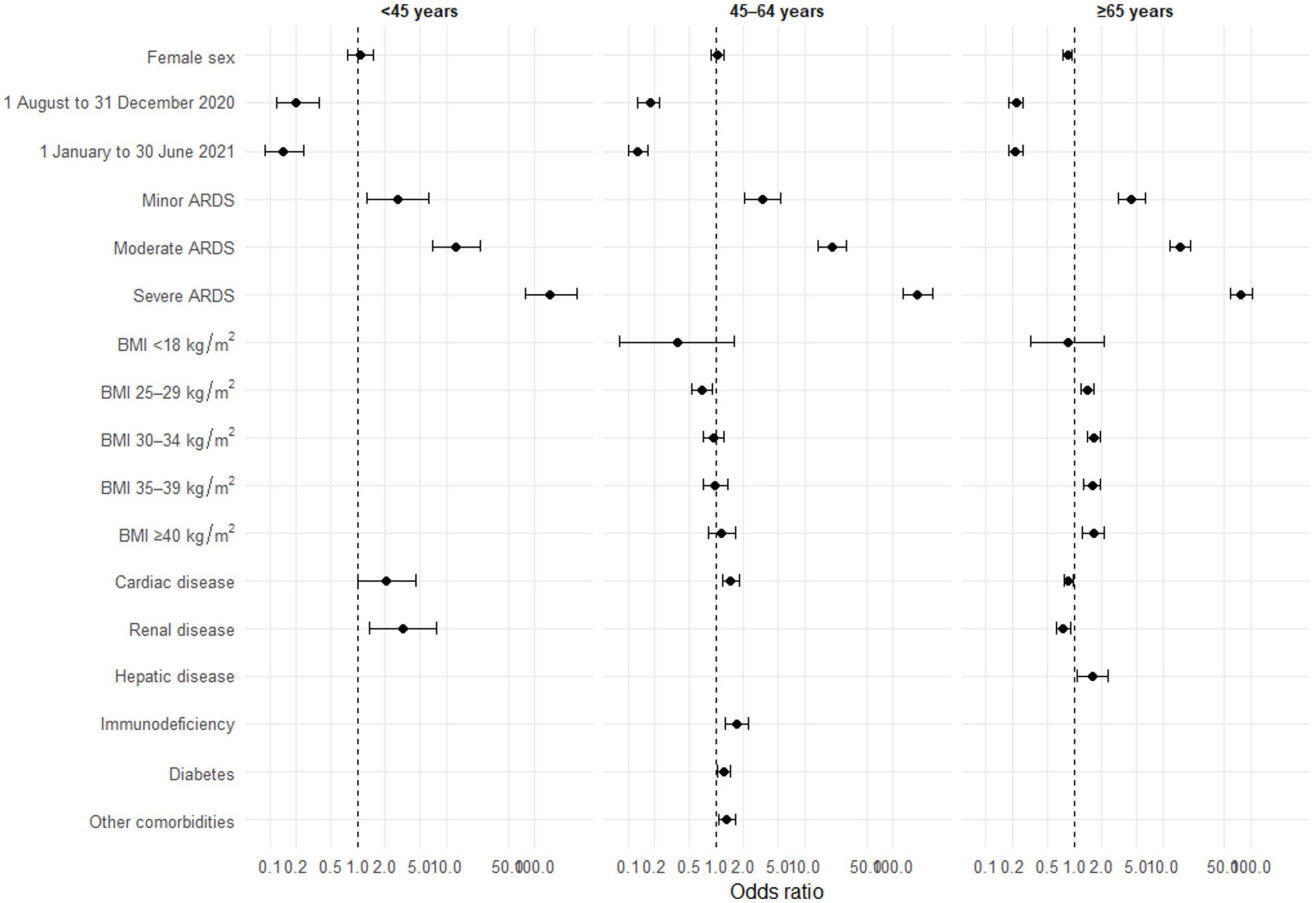
Forest plot of factors associated with the use of invasive ventilatory support by age group (*n* = 14,607), multivariate analyses. ARDS, acute respiratory distress syndrome, BMI, body mass index. Models adjusted for number of reports per ICU and region of care. A patient may have several comorbidities. Some comorbidities are not presented, either because they were not significant in univariate analyses, or because they were not retained by the step-by-step algorithm. Missing categories are also not presented.

This analysis included 14,607 patients admitted to ICU (see Supplementary File 9). The proportion of patients requiring invasive respiratory support increased with age and over time. Univariate analyses are presented in Supplementary File 10. In multivariate analyses, pandemic periods were associated with a lower use of these procedures, regardless of age (Figure 4). The more severe the ARDS, the greater the risk of using invasive respiratory support, regardless of age. Overweight and obese patients aged ≥65 years underwent invasive respiratory support more often than those of normal weight. Conversely, overweight patients aged between 45 and 64 years underwent these procedures less often than those of normal weight; there was no significant association for patients <45 years. Previous cardiac disease was also associated with an increased need for invasive respiratory support in patients <65 years, but significantly less frequent need in patients aged ≥65 years. A higher use of invasive respiratory support was associated with immunodeficiency and diabetes in patients aged between 45 and 64 years. Renal disease was associated with a higher use of invasive respiratory support in those <45 years and a lower use in patients aged ≥65 years. Hepatic disease was associated with a higher use of invasive respiratory support in patients aged ≥65 years. The quality of the multivariate models is presented in Supplementary File 11.

## Discussion

Based on national data from the surveillance of patients admitted to ICU for COVID-19 in France (~15,000 patients before June 2021), mortality remained unchanged between the three consecutive pandemic periods. Although mortality was similar, the number of COVID-19 patients admitted to ICU was higher in the third period compared with other periods suggesting an improvement in medical care. However, this apparent stability in ICU mortality should be interpreted in light of the substantial changes in ICU management during the pandemic. ICU admission criteria, ventilation strategies, including the increasing use of high-flow nasal oxygen and non-invasive ventilation, and pharmacological treatments such as corticosteroids, evolved considerably over time. In addition, the circulation of different SARS-CoV-2 variants may also have influenced disease severity and outcomes (27, 28). Some factors potentially influencing patient outcomes, including changes in clinical management over time, were not available in our dataset and therefore could not be accounted for in the analyses. These changes may partly explain the relatively stable mortality observed across the three pandemic periods, consistent with findings from other studies examining temporal trends in ICU outcomes during the COVID-19 pandemic (29). Furthermore, this study highlighted that sex and comorbidities (between six and eight depending on the outcome) were associated with an unfavorable outcome among patients with COVID-19. Another key finding was that being overweight or obese (<40 kg/m^2^) was associated with a reduced risk of death in patients over 45 years of age.

The demographic characteristics of patients were typical for ICU admissions with the majority of individuals being male, aged ≥65 years and having one or more comorbidities (30). It is well known that compared to younger patients, elderly patients have a poorer outcome. Advanced age can increase ICU complications due to patients’ lower tolerance of protocol care on account of their comorbidities. In agreement with previous literature, we observed a differential effect of age and sex on ICU mortality (31–35) and length of ICU stay (36–38). French data analyzed during the first three periods by the Direction de la Recherche, des Etudes, de l’Evaluation et des Statistiques (DREES) (21) showed that critical care duration was higher in men. The fact that men aged ≥65 had a higher risk of death and fewer ICU-free days than women may be explained by the different distribution of comorbidities. In fact, men ≥65 years had significantly more cardiac, pulmonary and renal pathologies, immunodeficiency, diabetes as well as higher BMI than women. Regarding the risk of using invasive respiratory support, other studies also found that sex (39) and age (40) were associated with intubation. Several studies found a differential effect of age and sex on the risk of ARDS (41, 42).

Nearly half of patients admitted to ICU for COVID-19 required invasive respiratory support, 44% had severe ARDS and 22% died. The case fatality rate (CFR) seems below the European rate (43). The CFR increased with age and among those requiring invasive mechanical ventilation and decreased after the second period of the pandemic. This is probably due to insufficient knowledge of the disease at the beginning of the pandemic and the significant hospital burden of COVID-19 on ICUs.

The three contexts examined for each objective were region of ICU admission, number of reported cases per ICU and time (three consecutive pandemic periods in France). The description of patients over time (Table 2) confirms the importance of taking this variable into account: higher BMI, increased HBP, less frequent use of invasive respiratory support and more patients with severe ARDS. Additionally, during the second period, the average age of patients was lower than in the first two periods. The third period was associated with severe ARDS in patients ≥65 years, with increased ICU-free days in patients <45 years and ≥65 years and with a reduced use of invasive respiratory support in all age categories. The association with ICU-free days can be partly explained by an evolution in the management of COVID-19, described in the literature as the extension of corticosteroid therapy, use of tocilizumab, management of anticoagulation and less frequent use of OTI (44–46). The association with severe ARDS may be partly explained by a shift in the selection criteria for ICU admission toward more hypoxemic cases. In fact, the rapid increase of cases during the first period of the pandemic led to the saturation of hospital capacity, including ICUs, giving rise to the need to prioritize admissions.

The use of mechanical ventilation significantly increased the risk of death and reduced ICU-free days, regardless of age. Invasive ventilation has already been shown to increase the risk of death (30) and length of hospital stay (47). Other studies demonstrated that the reduction over time in the use of OTI (48, 49) shows the beneficial effect of delaying invasive ventilation on the outcome of patients with COVID-19; they instead recommend that non-invasive ventilation techniques such as high-flow nasal cannula (HFNC) therapy should be favored as first-line treatment (50, 51). Respiratory assistance by ECMO was used less frequently and its use remained stable overall, regardless of the epidemic period.

Diabetes, hypertension, cardiac disease and obesity are recognized risk factors for developing severe forms of COVID-19 (52–55). A meta-analysis of 32 articles further identified the following diseases: liver disease, lung disease, malignant tumors, cerebrovascular disease, chronic obstructive pulmonary disease and asthma. Among all the underlying diseases, HBP had the highest prevalence, estimated at 46% (37–55%) (56). In our study, only cardiac disease had an impact on the four markers of severity depending on age, being associated with a higher risk of severe ARDS in individuals aged <45 years and a higher use of invasive respiratory support in those aged <65 years. By contrast, pulmonary diseases had no significant effect on the risk of severe ARDS or on the use of invasive respiratory support, although the risk of death in patients ≥45 years was higher. In individuals aged ≥65, cardiac and renal disease were associated with a lower use of invasive respiratory support. This result may be explained by the fact that these patients are often the most severe cases, leading to limited care. Being overweight and obesity were associated with lower mortality in patients aged ≥45 years, although this finding should be interpreted with caution and remains debated in the literature (57–61). Several mechanisms may explain this observation. First, differences in ICU admission practices may contribute, as patients with obesity may be admitted earlier or at a lower threshold because of concerns regarding rapid respiratory deterioration. Second, residual confounding common in observational studies can play a role. In particular, our dataset did not include frailty or sarcopenia, which are more frequent in older individuals with lower BMI and may be associated with poorer outcomes (62, 63). In this context, lower BMI may partly reflect underlying vulnerability rather than a healthier baseline status. Third, differences in the management of severe respiratory failure may also contribute. For example, patients with obesity may be more frequently managed with prone positioning or other ventilatory strategies that could influence outcomes (64). In addition, limitations of life-sustaining treatments may also affect mortality estimates, as such decisions depend both on patient characteristics and on local ICU (65). Finally, interpretation of this association should take into account the proportion of missing BMI data (nearly 20%) in our cohort and the possibility of methodological effects such as residual confounding or selection mechanisms related to ICU admission, which have been proposed as explanations for the so-called “obesity paradox” reported in critically ill populations. On another note, obesity was associated with an increased risk of developing severe ARDS independently of age, with the use of invasive respiratory support in patients ≥65 years, but almost had no impact on ICU-free days. This has also been reported in other studies (66, 67).

As this study is observational, the associations reported are potentially subject to confounding, selection bias and measurement error. Nevertheless, surveillance data on COVID-19 patients admitted to ICU illustrate the dynamics of the pandemic: a first peak in critical care admissions in March 2020, a second peak in October 2020 and a third peak responsible for a sustained high number of monthly ICU admissions (21). Our study is limited to variables routinely collected in this national surveillance system. This surveillance system was based on the existing sentinel surveillance system for influenza. Data collected were restricted to a one-page standardized notification form which was forwarded to the Santé publique France regional offices. Several variables known to influence outcomes in critically ill COVID-19 (in particular, widely used severity scores such as the SOFA score, SAPS II or APACHE II) were not recorded. Similarly, detailed information on ventilatory parameters, respiratory support before ICU admission, timing of intubation, and the use of specific therapies (e.g., corticosteroids, immunomodulatory treatments or anticoagulation) were not routinely recorded. Furthermore, the French vaccination campaign started in December 2020, first targeting high-risk populations such as the elderly people in nursing homes and healthcare workers (68). It was then progressively extended to larger groups of the population, to finally target the whole adult population in May 2021 (27). This timeframe does not allow us to assess the effect of vaccination on ICU outcomes. Even in the absence of data on vaccination status, we can suggest that despite the increase in vaccination rates from January 2021 onwards, this did not translate into a decrease in mortality rates among COVID-19 patients, who were admitted to ICU (27). On the other hand, the Alpha variant, the main circulating variant, was then replaced by the Delta variant between August and December 2021 (69). Omicron BA.1 emerged in November 2021 and quickly replaced Delta in January 2022. Our 3 time periods did not necessarily coincide with the dominant variants, but they were close. Information about the variant of concern identified for each COVID-19 patient was not routinely recorded. These factors have been shown to strongly influence outcomes in ICU patients with COVID-19 (70, 71). For instance, a previous study has highlighted the importance of ventilatory strategies and the timing of non-invasive ventilation in determining clinical outcomes among patients with COVID-19-related acute respiratory distress syndrome (71). The absence of these variables may therefore result in residual confounding and may partly influence the associations observed in our analyses, particularly those related to mortality. Another limitation relates to the outcome definition. Only mortality occurring during ICU stay was available in the surveillance database. Information on in-hospital mortality after ICU discharge or on 30-day mortality was not available. Consequently, ICU mortality may underestimate the overall burden of disease, as some patients may die after transfer to other hospital wards following ICU discharge. ICU mortality should be interpreted as mortality occurring during the critical care phase rather than overall hospital mortality. Data on lifestyle factors known to influence the degree of ARDS, such as smoking, alcohol consumption and physical activity (41, 72), were not available. The collection of comorbidities was relatively imprecise based on groups of pathologies, which had the disadvantage of regrouping diseases of different severities on the same level. The presence of missing data, notably for BMI, ARDS and ventilation mode, also represented a limitation. We minimized this bias by including them in our analysis. The level of ARDS and the mode of ventilation were entered at discharge, which may partly explain the high proportion of missing data; only the maximum level reached during the stay was provided without detailing their evolution. In addition, patients with missing vital status or missing major comorbidities were excluded from the analyses (approximately 12% of the initial cohort). Because the excluded patients were younger and had fewer recorded comorbidities, their exclusion may have introduced selection bias, potentially leading to a slight overestimation of mortality and severity outcomes in the analyzed cohort. However, these exclusions were made to ensure the reliability of the analyses, particularly for the assessment of comorbidities and the primary outcome. On the other hand, clinicians are not unanimous regarding the definition of ARDS according to the Berlin definition of ARDS (73–75), which may lead to imprecise data or even bias in differential classification. This definition specifies that ARDS must be measured under mechanical ventilation. Thus, some clinicians consider that a patient on HFNC therapy does not have ARDS, whereas for others, a ventilated patient (whether invasive or not) in ICU has ARDS. Another limitation of our work is the heterogeneous distribution of severity indicators within the same ICU and between ICUs, inherent to the variability of patients, clinicians’ practices and facilities’ structures, although we partially took this effect into account in each analysis by adjusting for the number of reports per department and the region of care. Variations in the management of COVID-19 patients, such as the timing and application of mechanical ventilation and administration of medications, were not fully standardized between the different ICUs. In addition, we considered the region of care instead of patients’ region of residence, which enables us to better approximate the real impact of the pandemic on the hospital system despite the transfer of patients from one region to another. Furthermore, as shown in the results, the number of deaths was low among patients younger than 45 years (*n* = 58). This may have led to limited statistical power and possible overfitting, which could explain why we did not find any effect of the pandemic periods on mortality in this age group.

Strengths of this study were its follow-up time (throughout the three waves from the start of the pandemic), stratification according to age, real-life evidence of changes in practices and the large number of patients. As the majority of ICUs previously participated in the surveillance of influenza cases admitted to ICU, this facilitated the implementation of COVID-19 surveillance.

## Conclusion

In conclusion, ICU mortality remained unchanged between the three pandemic periods, but our findings highlight obesity and male sex as key risk factors in the most severe forms of the disease, particularly in older patients. Lastly, the study showed a reduction in the use of invasive respiratory support after August 2020, irrespective of patient severity. As this ICU surveillance is maintained during winter seasons, it will be important to continue this work to incorporate other variables into the analysis such as patient vaccination status or SARS-CoV-2 variants. Further studies are needed to fully understand our observations as well as considering entire hospital stay (pre- and post-ICU).

## Data Availability

The datasets presented in this article are not readily available because the datasets generated and analyzed during the current study are not publicly available due to their pseudonymization [access to the datasets requires an authorization from the French Data Protection Authority (CNIL)] but are available from the corresponding author on reasonable request. Requests to access the datasets should be directed to antoine.journe@u-bourgogne.fr.
