# Supplementary material for: Factors associated with an unfavorable outcome according to age in patients with COVID-19 admitted to intensive care in mainland France during the first three periods of the pandemic: a nationwide cohort study
Source: Front Med (Lausanne). 2026 Apr 23;13:1816657. doi: 10.3389/fmed.2026.1816657 (PMC13149367; doi:10.3389/fmed.2026.1816657)
Supplement: Supplementary file 1 [file Supplementary_file_1.docx]

Additional File 1: Comparison of included versus excluded cases, mainland France, February 2020-June 2021

|  | **Overall**  (n=17,488)^1^ | **Excluded**  (n=2,065)^1^ | **Included**  (n=15,423)^1^ |
| --- | --- | --- | --- |
| Sex |  |  |  |
| Women | 5,275 (30) | 591 (29) | 4,684 (30) |
| Men | 12,202 (70) | 1,463 (71) | 10,739 (70) |
| Missing data | 11 (<0.1) | 11 (0.5) | 0 (0) |
| Number of reports per ICU | | |  |
| <50 | 1,045 (6.0) | 225 (11) | 820 (5.3) |
| 50-99 | 1,537 (8.8) | 259 (13) | 1,278 (8.3) |
| ≥100 | 14,906 (85) | 1,581 (77) | 13,325 (86) |
| Pandemic periods (ICU admission date) | | | |
| 23 February to 31 July 2020 | 4,041 (23) | 774 (37) | 3,267 (21) |
| 1 August to 31 December 2020 | 5,333 (30) | 473 (23) | 4,860 (32) |
| 1 January to 30 June 2021 | 8,114 (46) | 818 (40) | 7,296 (47) |
| Age group (in years) | | |  |
| <45 | 1,329 (7.6) | 192 (9.3) | 1,137 (7.4) |
| 45-64 | 6,174 (35) | 768 (37) | 5,406 (35) |
| ≥65 | 9,933 (57) | 1,053 (51) | 8,880 (58) |
| Missing data | 52 (0.3) | 52 (2.5) | 0 |
| Region of care | | |  |
| IDF | 1,035 (5.9) | 300 (15) | 735 (4.8) |
| ARA | 2,403 (14) | 574 (28) | 1,829 (12) |
| BFC | 1,207 (6.9) | 14 (0.7) | 1,193 (7.7) |
| BRE | 667 (3.8) | 84 (4.1) | 583 (3.8) |
| COR | 133 (0.8) | 7 (0.3) | 126 (0.8) |
| CVL | 728 (4.2) | 109 (5.3) | 619 (4.0) |
| GES | 276 (1.6) | 25 (1.2) | 251 (1.6) |
| HDF | 2,342 (13) | 367 (18) | 1,975 (13) |
| NAQ | 1,440 (8.2) | 167 (8.1) | 1,273 (8.3) |
| NOR | 1,000 (5.7) | 23 (1.1) | 977 (6.3) |
| OCC | 2,316 (13) | 72 (3.5) | 2,244 (15) |
| PACA | 2,083 (12) | 69 (3.3) | 2,014 (13) |
| PDL | 1,858 (11) | 254 (12) | 1,604 (10) |
| Maximum ventilatory support achieved during stay |  |  |  |
| Neither OTI nor ECMO | 7,925 (45) | 881 (43) | 7,044 (46) |
| OTI and/or ECMO | 8,430 (48) | 867 (42) | 7,563 (49) |
| Missing data | 1,133 (7) | 317 (15) | 816 (5) |
| Maximum ARDS reached during stay | | |  |
| Absence | 2,404 (14) | 281 (14) | 2,123 (14) |
| Minor | 1,191 (6.8) | 138 (6.7) | 1,053 (6.8) |
| Moderate | 4,508 (26) | 384 (19) | 4,124 (27) |
| Severe | 7,292 (42) | 558 (27) | 6,734 (44) |
| Missing data | 2,093 (12) | 704 (34) | 1,389 (9.0) |
| BMI by class (in kg/m^2^) | | |  |
| <18 | 52 (0.3) | 4 (0.2) | 48 (0.3) |
| 18-24 | 2,185 (12) | 230 (11) | 1,955 (13) |
| 25-29 | 4,895 (28) | 579 (28) | 4,316 (28) |
| 30-34 | 3,779 (22) | 260 (13) | 3,519 (23) |
| 35-39 | 1,749 (10) | 114 (5.5) | 1,635 (11) |
| ≥40 | 1,134 (6.5) | 93 (4.5) | 1,041 (6.7) |
| Missing data | 3,694 (21) | 785 (38) | 2,909 (19) |
| Cardiac disease | 3,608 (21) | 279 (14) | 3,329 (22) |
| Pulmonary disease | 3,336 (19) | 264 (13) | 3,072 (20) |
| Renal disease | 1,214 (6.9) | 80 (3.9) | 1,134 (7.4) |
| Hepatic disease | 306 (1.7) | 17 (0.8) | 289 (1.9) |
| Neuromuscular disease | 521 (3.0) | 44 (2.1) | 477 (3.1) |
| Cancer | 841 (4.8) | 54 (2.6) | 787 (5.1) |
| Immunodeficiency | 1,118 (6.4) | 91 (4.4) | 1,027 (6.7) |
| Diabetes (types 1 and 2) | 4,708 (27) | 391 (19) | 4,317 (28) |
| High blood pressure | 6,997 (40) | 506 (25) | 6,491 (42) |
| Other comorbidities | 2,176 (12) | 133 (6.4) | 2,043 (13) |
| Evolution |  |  |  |
| No information | 1,288 (7.4) | 1,288 (62) | 0 |
| Death | 3,579 (20) | 128 (6.2) | 3,451 (22) |
| Transfer to another ICU | 469 (2.7) | 44 (2.1) | 425 (2.8) |
| Transfer out of ICU or hospital discharge | 12,152 (69) | 605 (29) | 11,547 (75) |
| Length of ICU stay (in days) | 10 (5, 21) | 9 (4, 19) | 10 (5, 21) |
| Missing data | 1500 (8.6) | 1500 (73) | 0 |

^1^ n (%), Median (IQR)

Abbreviations:

ARA: Auvergne-Rhône-Alpes, ARDS: acute respiratory distress syndrome, BFC: Bourgogne-Franche-Comté, BMI: body mass index, BRE: Bretagne, COR: Corse, CVL: Centre-Val de Loire, ECMO: extracorporeal membrane oxygenation, GES: Grand Est, HDF: Hauts-de-France, ICU: intensive care unit, IDF: Île-de-France, NAQ: Nouvelle-Aquitaine, NOR: Normandie, OCC: Occitanie, OTI: orotracheal intubation, PACA: Provence-Alpes-Côte d’Azur, PDL: Pays de la Loire

Reading note:

A patient may have several comorbidities.
