# Supplementary material for: Factors associated with an unfavorable outcome according to age in patients with COVID-19 admitted to intensive care in mainland France during the first three periods of the pandemic: a nationwide cohort study
Source: Front Med (Lausanne). 2026 Apr 23;13:1816657. doi: 10.3389/fmed.2026.1816657 (PMC13149367; doi:10.3389/fmed.2026.1816657)
Supplement: Supplementary file 2 [file Supplementary_file_2.docx]

Additional File 2: Factors associated with mortality by age group (n=15,423), mainland France, February 2020-June 2021, univariate analyses

|  | **<45 years**  (n=1,137) | | **45-64 years**  (n=5,406) | | | **≥65 years**  (n=8,880) | |
| --- | --- | --- | --- | --- | --- | --- | --- |
|  | *SHR*  *(95% CI)^1^* | *p-value^1^* | *SHR*  *(95% CI)^1^* | *p-value^1^* | *SHR*  *(95% CI)^1^* | | *p-value^1^* |
| Female sex | 0.90  (0.52 – 1.53) | 0.69 | 0.88  (0.74 – 1.05) | 0.16 | 0.77  (0.10 – 0.83) | | <0.001 |
| Number of reports per ICU |  | 0.40 |  | 0.09 |  | | 0.25 |
| <50 | 1.63  (0.80 – 3.30) | 0.18 | 1.36  (0.97 – 1.89) | 0.07 | 1.12  (0.95 – 1.32) | | 0.19 |
| 50-99 | 1.03  (0.37 – 2.86) | 0.95 | 1.21  (0.93 – 1.58) | 0.16 | 1.08  (0.95 - 1.22) | | 0.27 |
| ≥100 | Ref | Ref | Ref | Ref | Ref | | Ref |
| Pandemic periods (ICU admission date) |  | 0.01 |  | 0.002 |  | | 0.19 |
| 23 February to 31 July 2020 | Ref | Ref | Ref | Ref | Ref | | Ref |
| 1 August to 31 December 2020 | 1.21  (0.64 – 2.29) | 0.56 | 1.10  (0.89 – 1.37) | 0.36 | 1.06  (0.95 – 1.17) | | 0.28 |
| 1 January to 30 June 2021 | 0.50  (0.26 – 0.95) | 0.04 | 0.81  (0.66 – 0.99) | 0.04 | 0.98  (0.89 – 1.08) | | 0.70 |
| Maximum ventilatory support achieved during stay |  | <0.001 |  | <0.001 |  | | <0.001 |
| Neither OTI nor ECMO | Ref | Ref | Ref | Ref | Ref | | Ref |
| OTI and/or ECMO | 9.95  (4.70 – 21.07) | <0.001 | 7.50  (5.93 – 9.47) | <0.001 | 2.38  (2.18 – 2.60) | | <0.001 |
| Missing data | 1.82  (0.39 – 8.56) | <0.001 | 3.38  (2.23 – 5.14) | <0.001 | 1.34  (1.10 – 1.63) | | 0.004 |
| Maximum ARDS reached during stay |  | <0.001 |  | <0.001 |  | | <0.001 |
| Absence | Ref | Ref | Ref | Ref | Ref | | Ref |
| Minor | 0.80  (0.17 – 3.87) | <0.001 | 0.70  (0.34 – 1.45) | <0.001 | 0.95  (0.68 – 1.31) | | 0.73 |
| Moderate | 0.55  (0.16 – 1.88) | <0.001 | 1.31  (0.83 – 2.07) | <0.001 | 1.17  (0.94 – 1.46) | | 0.15 |
| Severe | 5.74  (2.56 – 12.86) | <0.001 | 7.67  (5.15 – 11.43) | <0.001 | 5.09  (4.19 – 6.18) | | <0.001 |
| Missing data | 2.85  (0.96 – 8.44) | <0.001 | 3.43  (2.14 – 5.48) | <0.001 | 2.81  (2.23 – 3.52) | | <0.001 |
| BMI by class (in kg/m^2^) |  | 0.07 |  | <0.001 |  | | <0.001 |
| <18 | 11.78  (1.87 – 74.03) | 0.009 | 1.53  (0.47 – 4.95) | 0.48 | 1.64  (0.88 – 3.06) | | 0.12 |
| 18-24 | Ref | Ref | Ref | Ref | Ref | | Ref |
| 25-29 | 1.56  (0.42 – 5.75) | 0.51 | 0.56  (0.44 – 0.73) | <0.001 | 0.83  (0.74 – 0.93) | | 0.001 |
| 30-34 | 2.09  (0.61 – 7.17) | 0.24 | 0.47  (0.36 -0.62) | <0.001 | 0.83  (0.74 – 0.94) | | 0.003 |
| 35-39 | 0.64  (0.13 – 3.20) | 0.53 | 0.67  (0.50 – 0.90) | <0.001 | 0.82  (0.70 – 0.96) | | 0.01 |
| ≥40 | 2.16  (0.61 – 7.64) | 0.23 | 0.83  (0.61 – 1.14) | 0.25 | 0.83  (0.67 – 1.03) | | 0.09 |
| Missing data | 2.06  (0.59 – 7.24) | 0.26 | 0.77  (0.59 – 1.01) | 0.06 | 1.00  (0.88 - 1.13) | | 0.95 |
| Cardiac diseases | 3.27  (1.60 – 6.67) | 0.001 | 2.39  (1.98 – 2.87) | <0.001 | 1.46  (1.35 – 1.58) | | <0.001 |
| Pulmonary diseases | 1.91  (1.03 – 3.54) | 0.04 | 1.52  (1.27 – 1.83) | <0.001 | 1.35  (1.24 – 1.47) | | <0.001 |
| Renal diseases | 3.97  (1.99 – 7.93) | <0.001 | 2.91  (2.28 – 3.71) | <0.001 | 1.77  (1.59 – 1.98) | | <0.001 |
| Hepatic diseases | 5.33  (1.64 – 17.30) | 0.005 | 3.36  (2.43 – 4.66) | <0.001 | 1.94  (1.56 – 2.41) | | <0.001 |
| Neuromuscular diseases | 2.97  (1.04 – 8.43) | 0.04 | 1.70  (1.13 – 2.56) | 0.01 | 1.61  (1.35 – 1.93) | | <0.001 |
| Cancer | 4.10  (1.54 – 10.90) | 0.005 | 2.90  (2.23 – 3.78) | <0.001 | 1.66  (1.46 – 1.89) | | <0.001 |
| Immunodeficiency | 2.48  (1.91 – 5.18) | 0.02 | 3.27  (2.67 - 4.00) | <0.001 | 1.66  (1.46 – 1.88) | | <0.001 |
| Diabetes (types 1 and 2) | 2.14  (1.16 – 3.96) | 0.02 | 1.29  (1.09 – 1.53) | 0.004 | 1.15  (1.06 – 1.24) | | <0.001 |
| High blood pressure | 3.23  (1.86 – 5.61) | <0.001 | 1.21  (1.03 – 1.43) | 0.02 | 1.06  (0.98 – 1.14) | | 0.15 |
| Other comorbidities | 1.49  (0.80 – 2.77) | 0.21 | 1.27  (1.03 – 1.57) | 0.03 | 1.21  (1.09 – 1.34) | | <0.001 |

^1^ Fine and Gray’s competitive risk model

Abbreviations:

ARDS: acute respiratory distress syndrome, BMI: body mass index, ECMO: extracorporeal membrane oxygenation, ICU: intensive care unit, OTI: orotracheal intubation, Ref: reference class, SHR: subdistribution hazard ratio, 95% CI: 95% confidence interval

Reading notes:

A patient may have several comorbidities.
