# Supplementary material for: Factors associated with an unfavorable outcome according to age in patients with COVID-19 admitted to intensive care in mainland France during the first three periods of the pandemic: a nationwide cohort study
Source: Front Med (Lausanne). 2026 Apr 23;13:1816657. doi: 10.3389/fmed.2026.1816657 (PMC13149367; doi:10.3389/fmed.2026.1816657)

Additional File 3: H-H Plots, multivariate mortality models

Additional File 3.1: Age <45 years


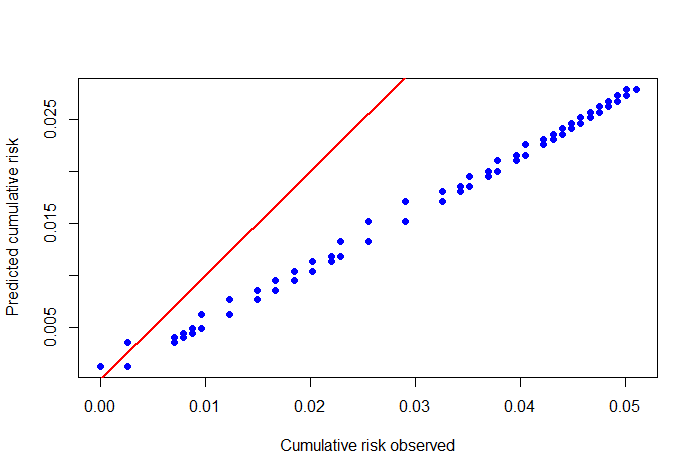


Additional File 3.2: Age 45-64 years


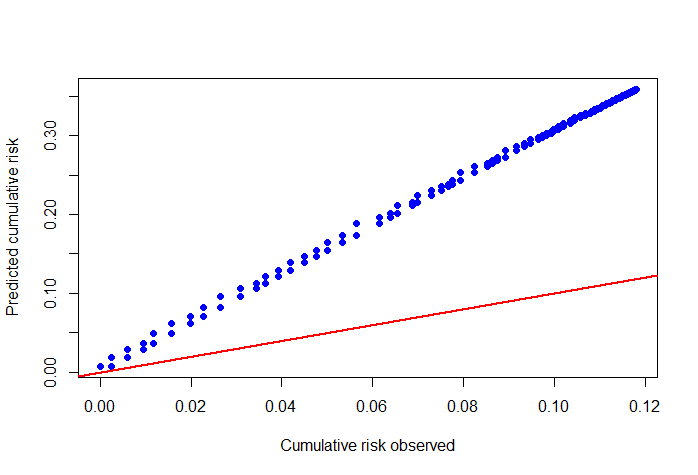


Additional File 3.3: Age ≥65 years


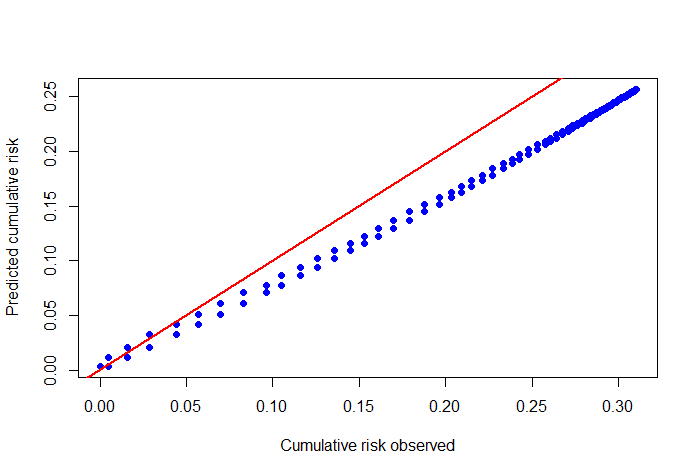

Supplement: Supplementary file 3 [file Supplementary_file_3.docx]
