# Supplementary material for: Factors associated with an unfavorable outcome according to age in patients with COVID-19 admitted to intensive care in mainland France during the first three periods of the pandemic: a nationwide cohort study
Source: Front Med (Lausanne). 2026 Apr 23;13:1816657. doi: 10.3389/fmed.2026.1816657 (PMC13149367; doi:10.3389/fmed.2026.1816657)
Supplement: Supplementary file 4 [file Supplementary_file_4.docx]

Additional File 4: Description of patients with COVID-19 admitted to intensive care, severe versus absence/mild/moderate ARDS model, mainland France, February 2020-June 2021

|  | **Overall**  (n=14,034)^1^ | **From absence to moderate ARDS**  (n=7,300)^1^ | **Severe ARDS**  (n=6,734)^1^ |
| --- | --- | --- | --- |
| Sex |  |  |  |
| Female | 4.241 (30) | 2,350 (32) | 1,891 (28) |
| Male | 9,793 (70) | 4,950 (68) | 4,843 (72) |
| Age group (in years) | | |  |
| <45 | 1,044 (7.4) | 732 (10) | 312 (4.6) |
| 45-64 | 4,893 (35) | 2,769 (38) | 2,124 (32) |
| ≥65 | 8,097 (58) | 3,799 (52) | 4,298 (64) |
| Number of reports per ICU | | |  |
| <50 | 743 (5.3) | 389 (5.3) | 354 (5.3) |
| 50-99 | 1,144 (8.2) | 563 (7.7) | 581 (8.6) |
| ≥100 | 12,147 (87) | 6,348 (87) | 5,799 (86) |
| Pandemic periods (ICU admission date) | | | |
| 23 February to 31 July 2020 | 3,156 (22) | 1,772 (24) | 1,384 (21) |
| 1 August to 31 December 2020 | 4,271 (30) | 2,211 (30) | 2,060 (31) |
| 1 January to 30 June 2021 | 6,607 (47) | 3,317 (45) | 3,290 (49) |
| Region of care | | |  |
| IDF | 695 (5.0) | 327 (4.5) | 368 (5.5) |
| ARA | 1,611 (11) | 822 (11) | 789 (12) |
| BFC | 1,170 (8.3) | 544 (7.5) | 626 (9.3) |
| BRE | 424 (3.0) | 239 (3.3) | 185 (2.7) |
| COR | 124 (0.9) | 57 (0.8) | 67 (1.0) |
| CVL | 594 (4.2) | 412 (5.6) | 182 (2.7) |
| GES | 240 (1.7) | 89 (1.2) | 151 (2.2) |
| HDF | 1,505 (11) | 804 (11) | 701 (10) |
| NAQ | 1,187 (8.5) | 584 (8.0) | 603 (9.0) |
| NOR | 923 (6.6) | 557 (7.6) | 366 (5.4) |
| OCC | 2,026 (14) | 967 (13) | 1,059 (16) |
| PACA | 1,975 (14) | 969 (13) | 1,006 (15) |
| PDL | 1,560 (11) | 929 (13) | 631 (9.4) |
| BMI by class (in kg/m^2^) | | |  |
| <18 | 44 (0.3) | 29 (0.4) | 15 (0.2) |
| 18-24 | 1,789 (13) | 956 (13) | 833 (12) |
| 25-29 | 3,963 (28) | 2,113 (29) | 1,850 (27) |
| 30-34 | 1,466 (10) | 655 (9.0) | 811 (12) |
| 35-39 | 1,244 (8.9) | 554 (7.6) | 690 (10) |
| ≥40 | 927 (6.6) | 418 (5.7) | 509 (7.6) |
| Missing data | 2,677 (19) | 1,541 (21) | 1,136 (17) |
| Cardiac diseases | 3,039 (22) | 1,443 (20) | 1,596 (24) |
| Pulmonary diseases | 2,785 (20) | 1,387 (19) | 1,398 (21) |
| Renal diseases | 1,030 (7.3) | 516 (7.1) | 514 (7.6) |
| Hepatic diseases | 262 (1.9) | 112 (1.5) | 150 (2.2) |
| Neuromuscular diseases | 437 (3.1) | 219 (3.0) | 218 (3.2) |
| Cancer | 698 (5.0) | 288 (3.9) | 410 (6.1) |
| Immunodeficiency | 916 (6.5) | 427 (5.8) | 489 (7.3) |
| Diabetes (types 1 and 2) | 3,895 (28) | 1,863 (26) | 2,032 (30) |
| High blood pressure | 5,873 (42) | 2,747 (38) | 3,126 (46) |
| Other comorbidities | 1,803 (13) | 886 (12) | 917 (14) |
| Evolution |  |  |  |
| Death | 3,159 (23) | 591 (8.1) | 2,568 (38) |
| Transfer out of or to another ICU, or hospital discharge | 10,875 (77) | 6,709 (92) | 4,166 (62) |
| Length of ICU stay (in days) | 10 (5 - 21) | 6 (3 - 11) | 17 (9 - 30) |

^1^ n (%), Median (IQR)

Abbreviations:

ARA: Auvergne-Rhône-Alpes, ARDS: acute respiratory distress syndrome, BFC: Bourgogne-Franche-Comté, BMI: body mass index, BRE: Bretagne, COR: Corse, CVL: Centre-Val de Loire, GES: Grand Est, HDF: Hauts-de-France, ICU: intensive care unit, IDF: Île-de-France, NAQ: Nouvelle-Aquitaine, NOR: Normandie, OCC: Occitanie, PACA: Provence-Alpes-Côte d’Azur, PDL: Pays de la Loire

Reading notes:

A patient may have several comorbidities.
