# Supplementary material for: Factors associated with an unfavorable outcome according to age in patients with COVID-19 admitted to intensive care in mainland France during the first three periods of the pandemic: a nationwide cohort study
Source: Front Med (Lausanne). 2026 Apr 23;13:1816657. doi: 10.3389/fmed.2026.1816657 (PMC13149367; doi:10.3389/fmed.2026.1816657)
Supplement: Supplementary file 5 [file Supplementary_file_5.docx]

Additional File 5: Factors associated with the risk of severe versus absence/mild/moderate ARDS by age class (n=14,034), mainland France, February 2020-June 2021, univariate analyses

|  | **<45 years**  (n=1,044) | | **45-64 years**  (n=4,893) | | **≥65 years**  (n=8,097) | |
| --- | --- | --- | --- | --- | --- | --- |
|  | *OR (95% CI)^1^* | *p-value^1^* | *OR (95% CI)^1^* | *p-value^1^* | *OR (95% CI)^1^* | *p-value^1^* |
| Female sex | 0.90 (0.68 – 1.18) | 0.45 | 0.82 (0.72 – 0.92) | 0.002 | 0.84 (0.76 – 0.92) | <0.001 |
| Number of reports per ICU |  | 0.05 |  | 0.17 |  | 0.21 |
| <50 | 0.55 (0.32 – 0.88) | 0.02 | 1.25 (0.96 – 1.63) | 0.10 | 1.07 (0.88 – 1.31) | 0.50 |
| 50-99 | 0.96 (0.56 – 1.59) | 0.86 | 1.11 (0.90 – 1.37) | 0.31 | 1.14 (0.98 – 1.34) | 0.10 |
| ≥100 | Ref | Ref | Ref | Ref | Ref | Ref |
| Region of care |  | 0.003 |  | <0.001 |  | <0.001 |
| ARA | 0.54 (0.30 – 0.97) | 0.04 | 0.71 (0.53 – 0.95) | 0.02 | 0.95 (0.74 – 1.22) | 0.69 |
| BFC | 0.65 (0.33 – 1.24) | 0.19 | 0.75 (0.54 – 1.03) | 0.08 | 1.13 (0.88 – 1.47) | 0.34 |
| BRE | 0.38 (0.15 – 0.89) | 0.03 | 0.45 (0.29 – 0.68) | <0.001 | 0.89 (0.64 – 1.23) | 0.48 |
| COR | 0.76 (0.15 – 3.31) | 0.72 | 0.80 (0.40 – 1.61) | 0.53 | 1.15 (0.70 – 1.89) | 0.58 |
| CVL | 0.54 (0.25 – 1.14) | 0.11 | 0.36 (0.25 – 0.52) | <0.001 | 0.38 (0.28 – 0.53) | <0.001 |
| GES | 1.27 (0.33 – 4.89) | 0.72 | 1.41 (0.88 – 2.27) | 0.16 | 1.51 (1.00 – 2.30) | 0.05 |
| HDF | 0.44 (0.24 – 0.80) | 0.007 | 0.70 (0.53 – 0.94) | 0.02 | 0.84 (0.65 – 1.09) | 0.19 |
| IDF | Ref | Ref | Ref | Ref | Ref | Ref |
| NAQ | 0.64 (0.34 – 1.18) | 0.15 | 0.74 (0.55 – 1.01) | 0.06 | 1.04 (0.80 – 1.36) | 0.75 |
| NOR | 0.41 (0.18 – 0.88) | 0.03 | 0.47 (0.34 – 0.65) | <0.001 | 0.64 (0.48 – 0.84) | 0.001 |
| OCC | 0.60 (0.34 – 1.05) | 0.07 | 0.77 (0.58 – 1.03) | 0.08 | 1.10 (0.86 – 1.41) | 0.43 |
| PACA | 0.64 (0.37 – 1.11) | 0.11 | 0.81 (0.61 – 1.07) | 0.14 | 1.01 (0.79 – 1.29) | 0.92 |
| PDL | 0.23 (0.12 – 0.43) | <0.001 | 0.52 (0.39 – 0.70) | <0.001 | 0.70 (0.55 – 0.91) | 0.007 |
| Pandemic periods (ICU admission date) |  | 0.09 |  | 0.30 |  | <0.001 |
| 23 February to 31 July 2020 | Ref | Ref | Ref | Ref | Ref | Ref |
| 1 August to 31 December 2020 | 0.91 (0.61 – 1.36) | 0.65 | 0.97 (0.83 – 1.15) | 0.76 | 1.27 (1.12 – 1.43) | <0.001 |
| 1 January to 30 June 2021 | 1.28 (0.93 – 1.79) | 0.13 | 1.08 (0.94 – 1.24) | 0.30 | 1.44 (1.29 – 1.62) | <0.001 |
| BMI by class (in kg/m^2^) |  | <0.001 |  | <0.001 |  | <0.001 |
| ≤18 | 1.06 (0.05 – 7.69) | 0.96 | 0.52 (0.11 – 1.75) | 0.33 | 0.65 (0.29 – 1.40) | 0.28 |
| 18-24 | Ref | Ref | Ref | Ref | Ref | Ref |
| 25-29 | 1.29 (0.70 – 2.43) | 0.42 | 1.03 (0.83 – 1.28) | 0.82 | 1.03 (0.90 – 1.18) | 0.68 |
| 30-34 | 2.82 (1.62 - 5.13) | <0.001 | 1.17 (0.94 – 1.46) | 0.16 | 1.21 (1.04 – 1.40) | 0.01 |
| 35-39 | 2.13 (1.16 – 4.06) | 0.02 | 1.76 (1.38 – 2.26) | <0.001 | 1.49 (1.24 – 1.79) | <0.001 |
| >40 | 2.77 (1.55 – 5.16) | <0.001 | 1.98 (1.51 – 2.60) | <0.001 | 1.48 (1.16 – 1.89) | 0.002 |
| Missing data | 0.94 (0.51 – 1.79) | 0.86 | 0.99 (0.78 – 1.24) | 0.91 | 0.86 (0.74 – 0.99) | 0.04 |
| Cardiac diseases | 2.09 (1.23 – 3.52) | 0.006 | 1.20 (1.01 – 1.43) | 0.04 | 1.05 (0.95 – 1.15) | 0.33 |
| Pulmonary diseases | 1.49 (1.02 – 2.16) | 0.04 | 1.02 (0.87 – 1.18) | 0.84 | 1.06 (0.96 – 1.18) | 0.27 |
| Renal diseases | 1.14 (0.59 – 2.11) | 0.68 | 1.15 (0.88 – 1.50) | 0.29 | 0.95 (0.82 – 1.10) | 0.51 |
| Hepatic diseases | 1.57 (0.40 – 5.54) | 0.49 | 1.68 (1.15 – 2.49) | 0.008 | 1.29 (0.93 - 1.82) | 0.13 |
| Neuromuscular diseases | 1.11 (0.48 – 2.43) | 0.79 | 1.25 (0.86 – 1.79) | 0.24 | 0.96 (0.76 – 1.21) | 0.73 |
| Cancer | 1.50 (0.55 – 3.86) | 0.40 | 1.66 (1.23 – 2.24) | <0.001 | 1.40 (1.16 – 1.67) | <0.001 |
| Immunodeficiency | 1.41 (0.82 - 2.39) | 0.20 | 1.22 (0.98 – 1.53) | 0.08 | 1.28 (1.07 – 1.53) | 0.008 |
| Diabetes (types 1 and 2) | 1.59 (1.06 - 2.37) | 0.02 | 1.25 (1.10 – 1.43) | <0.001 | 1.11 (1.01 – 1.22) | 0.04 |
| High blood pressure | 1.78 (1.20 – 2.62) | 0.004 | 1.44 (1.27 – 1.62) | <0.001 | 1.17 (1.07 -1.28) | <0.001 |
| Other comorbidities | 1.41 (0.99 – 1.99) | 0.06 | 1.04 (0.88 – 1.23) | 0.65 | 1.22 (1.07 - 1.40) | 0.003 |

^1^ Logistic regression

Abbreviations:

ARA: Auvergne-Rhône-Alpes, ARDS: acute respiratory distress syndrome, BFC: Bourgogne-Franche-Comté, BMI: body mass index, BRE: Bretagne, COR: Corse, CVL: Centre-Val de Loire, ECMO: extracorporeal membrane oxygenation, GES: Grand Est, HDF: Hauts-de-France, ICU: intensive care unit, IDF: Île-de-France, NAQ: Nouvelle-Aquitaine, NOR: Normandie, OCC: Occitanie, OR: odds ratio, OTI: orotracheal intubation, PACA: Provence-Alpes-Côte d’Azur, PDL: Pays de la Loire, Ref: reference class, 95% CI: 95% confidence interval

Reading notes:

A patient may have several comorbidities.
