# Supplementary material for: Factors associated with an unfavorable outcome according to age in patients with COVID-19 admitted to intensive care in mainland France during the first three periods of the pandemic: a nationwide cohort study
Source: Front Med (Lausanne). 2026 Apr 23;13:1816657. doi: 10.3389/fmed.2026.1816657 (PMC13149367; doi:10.3389/fmed.2026.1816657)
Supplement: Supplementary file 6 [file Supplementary_file_6.docx]

Additional File 6: Quality of the multivariate models on the risk of severe versus absence/minor/moderate ARDS

|  | **<45 years** | **45-64 years** | **≥65 years** |
| --- | --- | --- | --- |
| C-statistic (95% CI) | 0.69 (0.66 – 0.73) | 0.63 (0.61 – 0.64) | 0.61 (0.60 – 0.62) |
| Hosmer-Lemeshow test | 0.71 | 0.06 | 0.28 |

Abbreviations:

95% CI: 95% confidence interval
