# Supplementary material for: Factors associated with an unfavorable outcome according to age in patients with COVID-19 admitted to intensive care in mainland France during the first three periods of the pandemic: a nationwide cohort study
Source: Front Med (Lausanne). 2026 Apr 23;13:1816657. doi: 10.3389/fmed.2026.1816657 (PMC13149367; doi:10.3389/fmed.2026.1816657)
Supplement: Supplementary file 7 [file Supplementary_file_7.docx]

Additional File 7: Factors associated with ICU-free days by age group (n=15,423), mainland France, February 2020-June 2021, univariate analyses

|  | **<45 years**  (n=1,137) | | **45-64 years**  (n=5,406) | | | **≥65 years**  (n=8,880) | |
| --- | --- | --- | --- | --- | --- | --- | --- |
|  | *Beta*  *(95% CI)^1^* | *p-value^1^* | *Beta*  *(95% CI)^1^* | *p-value^1^* | *Beta*  *(95% CI)^1^* | | *p-value^1^* |
| Female sex | 1.17  (0.06 – 2.29) | 0.04 | 1.43  (0.80 – 2.06) | <0.001 | 2.64  (2.13 – 3.14) | | <0.001 |
| Number of reports per ICU |  | 0.10 |  | <0.001 |  | | <0.001 |
| <50 | -1.80  (-3.61 – 0.01) | 0.05 | -3.07  (-4.42 - -1.72) | <0.001 | -2.75  (-3.82 - -1.68) | | <0.001 |
| 50-99 | 0.81  (-1.39 – 2.93) | 0.46 | -1.35  (-2.40 - -0.31) | 0.01 | -1.47  (-2.31 - -0.63) | | <0.001 |
| ≥100 | Ref | Ref | Ref | Ref | Ref | | Ref |
| Region of care |  | 0.05 |  | 0.001 |  | | <0.001 |
| ARA | 1.27  (-1.26 – 3.80) | 0.33 | 2.72  (0.58 – 3.55) | 0.007 | 0.23  (-1.12 – 1.58) | | 0.74 |
| BFC | 0.42  (-2.49 – 3.32) | 0.78 | 0.46  (-1.24 – 2.15) | 0.60 | -0.13  (-1.54 – 1.28) | | 0.85 |
| BRE | 3.73  (0.50 – 6.95) | 0.02 | 3.08  (1.14 – 5.02) | 0.002 | 0.78  (-0.90 – 2.46) | | 0.36 |
| COR | -0.89  (-7.65 – 5.87) | 0.80 | 1.55  (-2.07 – 5.17) | 0.40 | 0.39  (-2.34 – 3.12) | | 0.78 |
| CVL | -2.28  (-5.59 – 1.03) | 0.18 | 2.12  (0.30 – 3.94) | 0.02 | 0.77  (-0.91 – 2.46) | | 0.37 |
| GES | 2.28  (-3.36 – 7.91) | 0.43 | 2.05  (-0.41 – 4.52) | 0.10 | 1.04  (-1.15 – 3.22) | | 0.35 |
| HDF | 1.78  (-0.70 – 4.27) | 0.16 | 1.80  (0.35 – 3.25) | 0.01 | 0.38  (-0.97 – 1.72) | | 0.58 |
| IDF | Ref | Ref | Ref | Ref | Ref | | Ref |
| NAQ | 2.04  (-0.74 – 4.82) | 0.15 | 2.53  (0.96 – 4.09) | 0.002 | 1.81  (0.38 – 3.23) | | 0.01 |
| NOR | 2.03  (-1.17 – 5.24) | 0.21 | 2.19  (0.53 – 3.84) | 0.01 | 0.86  (-0.63 – 2.35) | | 0.26 |
| OCC | 1.57  (-0.86 – 4.01) | 0.21 | 1.69  (0.24 – 3.14) | 0.02 | 0.63  (-0.69 – 1.95) | | 0.35 |
| PACA | 1.82  (-0.63 – 4.28) | 0.15 | 2.15  (0.70 – 3.59) | 0.004 | 0.58  (-0.77 – 1.92) | | 0.40 |
| PDL | 3.50  (0.98 – 6.02) | 0.01 | 3.48  (1.98 – 4.98) | <0.001 | 2.57  (1.19 – 3.96) | | <0.001 |
| Pandemic periods (ICU admission date) |  | <0.001 |  | <0.001 |  | | <0.001 |
| 23 February to 31 July 2020 | Ref | Ref | Ref | Ref | Ref | | Ref |
| 1 August to 31 December 2020 | 1.82  (0.23 – 3.40) | 0.02 | 2.17  (1.35 – 2.99) | <0.001 | 2.28  (1.63 – 2.92) | | <0.001 |
| 1 January to 30 June 2021 | 2.83  (1.48 – 4.18) | <0.001 | 3.12  (2.39 – 3.85) | <0.001 | 2.80  (2.18 – 3.42) | | <0.001 |
| Maximum ventilatory support achieved during stay |  | <0.001 |  | <0.001 |  | | <0.001 |
| Neither OTI nor ECMO | Ref | Ref | Ref | Ref | Ref | | Ref |
| OTI and/or ECMO | -12.14  (-13.06 - -11.21) | <0.001 | -13.68  (-14.14 - -13.21) | <0.001 | -11.75  (-12.17 - -11.33) | | <0.001 |
| Missing data | -2.33  (-3.98 - -0.67) | 0.006 | -4.70  (-5.71 - -3.69) | <0.001 | -5.28  (-6.24 - -4.31) | | <0.001 |
| Maximum ARDS reached during stay |  | <0.001 |  | <0.001 |  | | <0.001 |
| Absence | Ref | Ref | Ref | Ref | Ref | | Ref |
| Minor | 0.14  (-1.57 – 1.84) | 0.88 | -0.60  (-1.63 – 0.43) | 0.25 | -1.39  (-2.42 - -0.36) | | 0.008 |
| Moderate | -2.19  (-3.42 - -0.96) | <0.001 | -4.40  (-5.16 - -3.65) | <0.001 | -5.37  (-6.08 - -4.65) | | <0.001 |
| Severe | -12.56  (-13.79 - -11.33) | <0.001 | -15.01  (-15.73 - -14.29) | <0.001 | -15.06  (-15.72 - -14.40) | | <0.001 |
| Missing data | -5.73  (-7.54 - -3.92) | <0.001 | -7.67  (-8.65 - -6.68) | <0.001 | -10.40  (-11.30 - -9.50) | | <0.001 |
| BMI by class (in kg/m^2^) |  | 0.002 |  | <0.001 |  | | <0.001 |
| <18 | -5.69  (-12.81 – 1.44) | 0.12 | 1.56  (-4.56 – 7.68) | 0.62 | 0.32  (-3.82 – 4.45) | | 0.88 |
| 18-24 | Ref | Ref | Ref | Ref | Ref | | Ref |
| 25-29 | 0.02  (-2.20 – 2.25) | 0.98 | 1.60  (0.53 – 2.67) | 0.003 | 0.10  (-0.64 – 0.84) | | 0.79 |
| 30-34 | -1.33  (-3.46 – 0.79) | 0.22 | 0.58  (-0.50 – 1.66) | 0.29 | -0.82  (-1.61 - -0.03) | | 0.04 |
| 35-39 | -0.99  (-3.31 – 1.32) | 0.40 | -1.06  (-2.29 – -0.16) | 0.09 | -0.90  (-1.88 – -0.08) | | 0.07 |
| ≥40 | -3.56  (-5.79 - -1.32) | 0.002 | -1.70  (-3.03 - -0.36) | 0.01 | -1.44  (-2.72 - -0.16) | | 0.03 |
| Missing data | -0.20  (-2.38 – 1.99) | 0.86 | -0.31  (-1.46 – 0.83) | 0.59 | -1.32  (-2.12 - -0.52) | | 0.001 |
| Cardiac diseases | -5.55  (-7.89 - -3.21) | <0.001 | -2.94  (-3.82 - -2.06) | <0.001 | -1.64  (-2.15 - -1.13) | | <0.001 |
| Pulmonary diseases | -2.57  (-4.16 - -0.97) | 0.002 | -0.86  (-1.61 - -0.11) | 0.03 | -1.13  (-1.70 - -0.57) | | <0.001 |
| Renal diseases | -4.64  (-7.23 - -2.05) | <0.001 | -4.42  (-5.75 - -3.08) | <0.001 | -1.76  (-2.57 - -0.95) | | <0.001 |
| Hepatic diseases | -8.72  (-13.82 - -3.62) | <0.001 | -3.30  (-5.26 - 1.34) | <0.001 | -2.61  (-4.38 - -0.84) | | 0.004 |
| Neuromuscular diseases | -2.37  (-5.76 – 1.03) | 0.17 | -2.10  (-3.94 - -0.26) | 0.03 | -1.87  (-3.14 - -0.60) | | 0.004 |
| Cancer | -5.49  (-9.52 - -1.45) | 0.008 | -3.72  (-5.21 - 2.23) | <0.001 | -1.99  (-2.96 - -1.03) | | <0.001 |
| Immunodeficiency | -4.50  (-6.75 - -2.24) | <0.001 | -4.81  (-5.94 - -3.69) | <0.001 | -2.78  (-3.73 - -1.84) | | <0.001 |
| Diabetes (types 1 and 2) | -1.74  (-3.41 – -0.07) | 0.04 | -1.70  (-2.37 – -1.04) | <0.001 | -0.94  (-1.44 - -0.44) | | <0.001 |
| High blood pressure | -3.39  (-5.02 - -1.75) | <0.001 | -1.69  (-2.30 - -1.08) | <0.001 | -0.02  (-0.49 – 0.45) | | 0.93 |
| Other comorbidities | -1.88  (-3.34 - -0.43) | 0.01 | -1.55  (-2.39 - -0.70) | <0.001 | -0.56  (-1.26 – 0.14) | | 0.12 |

^1^ Linear regression

Abbreviations:

ARA: Auvergne-Rhône-Alpes, ARDS: acute respiratory distress syndrome, BFC: Bourgogne-Franche-Comté, BMI: body mass index, BRE: Bretagne, COR: Corse, CVL: Centre-Val de Loire, ECMO: extracorporeal membrane oxygenation, GES: Grand Est, HDF: Hauts-de-France, ICU: intensive care unit, IDF: Île-de-France, NAQ: Nouvelle-Aquitaine, NOR: Normandie, OCC: Occitanie, OTI: orotracheal intubation, PACA: Provence-Alpes-Côte d’Azur, PDL: Pays de la Loire, Ref: reference class, 95% CI: 95% confidence interval

Reading notes:

A patient may have several comorbidities.
