# Supplementary material for: Factors associated with an unfavorable outcome according to age in patients with COVID-19 admitted to intensive care in mainland France during the first three periods of the pandemic: a nationwide cohort study
Source: Front Med (Lausanne). 2026 Apr 23;13:1816657. doi: 10.3389/fmed.2026.1816657 (PMC13149367; doi:10.3389/fmed.2026.1816657)
Supplement: Supplementary file 9 [file Supplementary_file_9.docx]

Additional File 9: Description of patients with COVID-19 admitted to intensive care, invasive ventilatory support model, mainland France, February 2020-June 2021

|  | **Overall**  (n=14,607)^1^ | **Absence of invasive ventilatory support**  (n=7,044)^1^ | **Invasive ventilatory support**  (n=7,563)^1^ |
| --- | --- | --- | --- |
| Sex |  |  |  |
| Female | 4,438 (30) | 2,278 (32) | 2,160 (29) |
| Male | 10,169 (70) | 4,766 (68) | 5,403 (71) |
| Age group (in years) | | |  |
| <45 | 1,049 (7.2) | 642 (9.1) | 407 (5.4) |
| 45-64 | 5,111 (35) | 2,638 (37) | 2,473 (33) |
| ≥65 | 8,447 (58) | 3,764 (53) | 4,683 (62) |
| Number of reports per ICU | | |  |
| <50 | 734 (5.0) | 288 (4.1) | 446 (5.9) |
| 50-99 | 1,184 (8.1) | 533 (7.6) | 651 (8.6) |
| ≥100 | 12,689 (87) | 6,223 (88) | 6,466 (85) |
| Pandemic periods (ICU admission date) | | | |
| 23 February to 31 July 2020 | 3,016 (21) | 889 (13) | 2,127 (28) |
| 1 August to 31 December 2020 | 4,403 (30) | 2,271 (32) | 2,132 (28) |
| 1 January to 30 June 2021 | 7,188 (49) | 3,884 (55) | 3,304 (44) |
| Region of care | | |  |
| IDF | 717 (4.9) | 282 (4.0) | 435 (5.8) |
| ARA | 1,653 (11) | 766 (11) | 887 (12) |
| BFC | 1,179 (8.1) | 424 (6.0) | 755 (10.0) |
| BRE | 525 (3.6) | 249 (3.5) | 276 (3.6) |
| COR | 123 (0.8) | 73 (1.0) | 50 (0.7) |
| CVL | 598 (4.1) | 217 (3.1) | 381 (5.0) |
| GES | 235 (1.6) | 62 (0.9) | 173 (2.3) |
| HDF | 1,750 (12) | 994 (14) | 756 (10.0) |
| NAQ | 1,194 (8.2) | 552 (7.8) | 642 (8.5) |
| NOR | 956 (6.5) | 563 (8.0) | 393 (5.2) |
| OCC | 2,144 (15) | 1,099 (16) | 1,045 (14) |
| PACA | 2,006 (14) | 1,010 (14) | 996 (13) |
| PDL | 1,527 (10) | 753 (11) | 774 (10) |
| Maximum ARDS reached during stay | | |  |
| Absence | 1,900 (13) | 1,766 (25) | 134 (1.8) |
| Minor | 1,029 (7.0) | 855 (12) | 174 (2.3) |
| Moderate | 4,086 (28) | 2,388 (34) | 1,698 (22) |
| Severe | 6,673 (46) | 1,424 (20) | 5,249 (69) |
| Missing data | 919 (6.3) | 611 (8.7) | 308 (4.1) |
| BMI by class (in kg/m^2^) | | |  |
| <18 | 46 (0.3) | 30 (0.4) | 16 (0.2) |
| 18-24 | 1,867 (13) | 1,000 (14) | 867 (11) |
| 25-29 | 4,126 (28) | 2,154 (31) | 1,972 (26) |
| 30-34 | 3,364 (23) | 1,591 (23) | 1,773 (23) |
| 35-39 | 1,552 (11) | 706 (10) | 846 (11) |
| ≥40 | 991 (6.8) | 435 (6.2) | 556 (7.4) |
| Missing data | 2,661 (18) | 1,128 (16) | 1,533 (20) |
| Cardiac diseases | 3,168 (22) | 1,453 (21) | 1,715 (23) |
| Pulmonary disease | 2,918 (20) | 1,389 (20) | 1,529 (20) |
| Renal diseases | 1,078 (7.4) | 534 (7.6) | 544 (7.2) |
| Hepatic diseases | 274 (1.9) | 102 (1.4) | 172 (2.3) |
| Neuromuscular diseases | 454 (3.1) | 216 (3.1) | 238 (3.1) |
| Cancer | 741 (5.1) | 345 (4.9) | 396 (5.2) |
| Immunodeficiency | 964 (6.6) | 398 (5.7) | 566 (7.5) |
| Diabetes (types 1 and 2) | 4,060 (28) | 1,799 (26) | 2,261 (30) |
| High blood pressure | 6,213 (43) | 2,852 (40) | 3,361 (44) |
| Other comorbidities | 1,911 (13) | 903 (13) | 1,008 (13) |
| Evolution |  |  |  |
| Death | 3,309 (23) | 794 (11) | 2,515 (33) |
| Transfer out of or to another ICU, or hospital discharge | 11,298 (77) | 6,250 (89) | 5,048 (67) |
| Length of stay (in days) | 10 (5 - 21) | 5 (3 - 9) | 18 (10 - 31) |

^1^ n (%), Median (IQR)

Abbreviations:

ARA: Auvergne-Rhône-Alpes, ARDS: acute respiratory distress syndrome, BFC: Bourgogne-Franche-Comté, BMI: body mass index, BRE: Bretagne, COR: Corse, CVL: Centre-Val de Loire, ECMO: extracorporeal membrane oxygenation, GES: Grand Est, HDF: Hauts-de-France, ICU: intensive care unit, IDF: Île-de-France, NAQ: Nouvelle-Aquitaine, NOR: Normandie, OCC: Occitanie, OTI: orotracheal intubation, PACA: Provence-Alpes-Côte d’Azur, PDL: Pays de la Loire

Reading notes:

A patient may have several comorbidities.
