# Supplementary material for: Factors associated with an unfavorable outcome according to age in patients with COVID-19 admitted to intensive care in mainland France during the first three periods of the pandemic: a nationwide cohort study
Source: Front Med (Lausanne). 2026 Apr 23;13:1816657. doi: 10.3389/fmed.2026.1816657 (PMC13149367; doi:10.3389/fmed.2026.1816657)
Supplement: Supplementary file 10 [file Supplementary_file_10.docx]

Additional File 10: Factors associated with the use of invasive respiratory support by age group (n=14,607), mainland France, February 2020-June 2021, univariate analyses

|  | **<45 years**  (n=1,049) | | **45-64 years**  (n=5,111) | | **≥65 years**  (n=8,447) | |
| --- | --- | --- | --- | --- | --- | --- |
|  | *OR (95% CI)^1^* | *p-value^1^* | *OR (95% CI)^1^* | *p-value^1^* | *OR (95% CI)^1^* | *p-value^1^* |
| Female sex | 0.88 (0.67 – 1.13) | 0.31 | 0.90 (0.80 – 1.02) | 0.10 | 0.81 (0.74 – 0.89) | <0.001 |
| Number of reports per ICU |  | 0.31 |  | <0.001 |  | <0.001 |
| <50 | 1.37 (0.88 – 2.12) | 0.15 | 1.75 (1.33 – 2.31) | <0.001 | 1.46 (1.19 – 1.79) | <0.001 |
| 50-99 | 1.18 (0.73 – 1.91) | 0.49 | 1.15 (0.94 – 1.41) | 0.18 | 1.17 (1.01 – 1.37) | 0.04 |
| ≥100 | Ref | Ref | Ref | Ref | Ref | Ref |
| Region of care |  | <0.001 |  | <0.001 |  | <0.001 |
| ARA | 0.72 (0.41 – 1.28) | 0.27 | 0.61 (0.46 – 0.82) | <0.001 | 0.81 (0.63 – 1.04) | 0.10 |
| BFC | 0.80 (0.42 – 1.52) | 0.50 | 0.94 (0.68 – 1.31) | 0.72 | 1.25 (0.96 – 1.63) | 0.09 |
| BRE | 0.25 (0.10 – 0.57) | 0.002 | 0.51 (0.35 – 0.75) | <0.001 | 0.96 (0.70 – 1.32) | 0.82 |
| COR | 0.32 (0.04 – 1.47) | 0.18 | 0.28 (0.13 – 0.59) | <0.001 | 0.53 (0.32 – 0.86) | 0.01 |
| CVL | 1.63 (0.78 – 3.44) | 0.20 | 0.79 (0.56 – 1.12) | 0.19 | 1.39 (1.01 – 1.92) | 0.04 |
| GES | 1.91 (0.56 – 7.59) | 0.32 | 1.40 (0.84 – 2.38) | 0.21 | 2.03 (1.31 – 3.23) | <0.001 |
| HDF | 0.47 (0.27 – 0.83) | 0.009 | 0.41 (0.31 – 0.55) | <0.001 | 0.53 (0.41 – 0.68) | <0.001 |
| IDF | Ref | Ref | Ref | Ref | Ref | Ref |
| NAQ | 0.59 (0.32 – 1.09) | 0.10 | 0.69 (0.51 – 0.94) | 0.02 | 0.78 (0.60 – 1.02) | 0.07 |
| NOR | 0.34 (0.15– 0.71) | 0.005 | 0.34 (0.25 – 0.47) | <0.001 | 0.52 (0.39 – 0.68) | <0.001 |
| OCC | 0.66 (0.38 – 1.12) | 0.12 | 0.50 (0.38 – 0.66) | <0.001 | 0.66 (0.52 – 0.84) | <0.001 |
| PACA | 0.47 (0.27 – 0.80) | 0.006 | 0.55 (0.42 – 0.73) | <0.001 | 0.71 (0.55 – 0.90) | 0.006 |
| PDL | 0.49 (0.27 – 0.86) | 0.01 | 0.58 (0.43 – 0.77) | <0.001 | 0.73 (0.57 – 0.95) | 0.02 |
| Pandemic periods (ICU admission date) |  | <0.001 |  | <0.001 |  | <0.001 |
| 23 February to 31 July 2020 | Ref | Ref | Ref | Ref | Ref | Ref |
| 1 August to 31 December 2020 | 0.40 (0.27 – 0.58) | <0.001 | 0.36 (0.30 – 0.43) | <0.001 | 0.39 (0.34 – 0.44) | <0.001 |
| 1 January to 30 June 2021 | 0.38 (0.27 – 0.52) | <0.001 | 0.31 (0.27 – 0.36) | <0.001 | 0.39 (0.34 – 0.44) | <0.001 |
| Maximum ARDS reached during stay |  | <0.001 |  | <0.001 |  | <0.001 |
| Absence | Ref | Ref | Ref | Ref | Ref | Ref |
| Minor | 1.71 (0.83 – 3.42) | 0.13 | 2.20 (1.43 – 3.40) | <0.001 | 3.41 (2.48 – 4.72) | <0.001 |
| Moderate | 4.70 (2.91 – 7.91) | <0.001 | 9.81 (7.13 – 13.86) | <0.001 | 10.26 (8.00 – 13.36) | <0.001 |
| Severe | 44.44 (26.91 – 76.57) | <0.001 | 71.96 (52.19 – 101.89) | <0.001 | 40.89 (32.00 – 53.05) | <0.001 |
| Missing data | 4.10 (2.01 – 8.18) | <0.001 | 8.06 (5.52 – 11.97) | <0.001 | 6.44 (4.77 – 8.77) | <0.001 |
| BMI by class (in kg/m^2^) |  | 0.25 |  | <0.001 |  | <0.001 |
| <18 | 1.07 (0.14 – 5.78) | 0.94 | 0.59 (0.15 – 1.88) | 0.39 | 0.61 (0.27 – 1.31) | 0.22 |
| 18-24 | Ref | Ref | Ref | Ref | Ref | Ref |
| 25-29 | 1.07 (0.63 – 1.82) | 0.81 | 0.78 (0.64 – 0.96) | 0.02 | 1.24 (1.08 – 1.42) | 0.002 |
| 30-34 | 1.38 (0.84 - 2.31) | 0.20 | 1.07 (0.87 – 1.31) | 0.54 | 1.52 (1.32 – 1.76) | <0.001 |
| 35-39 | 1.34 (0.78 – 2.32) | 0.30 | 1.34 (1.06 – 1.70) | 0.01 | 1.55 (1.29 – 1.85) | <0.001 |
| ≥40 | 1.73 (1.03 – 2.94) | 0.04 | 1.62 (1.25 – 2.10) | <0.001 | 1.61 (1.27 – 2.04) | <0.001 |
| Missing data | 1.53 (0.91 – 2.61) | 0.11 | 1.46 (1.17 – 1.83) | <0.001 | 1.68 (1.45 – 1.96) | <0.001 |
| Cardiac diseases | 2.58 (1.53 – 4.42) | <0.001 | 1.44 (1.22 – 1.71) | <0.001 | 0.89 (0.81 – 0.98) | 0.02 |
| Pulmonary diseases | 1.27 (0.89 – 1.81) | 0.18 | 1.09 (0.94 – 1.26) | 0.24 | 0.93 (0.84 – 1.04) | 0.19 |
| Renal diseases | 2.30 (1.28 – 4.19) | 0.006 | 1.60 (1.23 – 2.08) | <0.001 | 0.69 (0.60 – 0.80) | <0.001 |
| Hepatic diseases | 2.23 (0.71 – 7.58) | 0.17 | 1.58 (1.08 – 2.33) | 0.02 | 1.54 (1.10 - 2.18) | 0.01 |
| Neuromuscular diseases | 1.38 (0.64 – 2.93) | 0.40 | 1.22 (0.85 – 1.74) | 0.28 | 0.89 (0.71 – 1.12) | 0.33 |
| Cancer | 1.43 (0.56 – 3.58) | 0.44 | 1.26 (0.94 – 1.68) | 0.12 | 0.92 (0.77 – 1.10) | 0.35 |
| Immunodeficiency | 2.06 (1.24 - 3.47) | 0.006 | 1.63 (1.30 – 2.03) | <0.001 | 1.14 (0.96 – 1.36) | 0.13 |
| Diabetes (types 1 and 2) | 1.53 (1.05 - 2.23) | 0.03 | 1.39 (1.22 – 1.58) | <0.001 | 1.07 (0.97 – 1.17) | 0.16 |
| High blood pressure | 1.61 (1.12 – 2.31) | 0.01 | 1.30 (1.15 – 1.46) | <0.001 | 0.94 (0.87 - 1.03) | 0.19 |
| Other comorbidities | 1.31 (0.94 – 1.82) | 0.11 | 1.14 (0.97 – 1.34) | 0.11 | 0.98 (0.86 - 1.12) | 0.77 |

^1^ Logistic regression

Abbreviations:

ARA: Auvergne-Rhône-Alpes, ARDS: acute respiratory distress syndrome, BFC: Bourgogne-Franche-Comté, BMI: body mass index, BRE: Bretagne, COR: Corse, CVL: Centre-Val de Loire, ECMO: extracorporeal membrane oxygenation, GES: Grand Est, HDF: Hauts-de-France, ICU: intensive care unit, IDF: Île-de-France, NAQ: Nouvelle-Aquitaine, NOR: Normandie, OCC: Occitanie, OTI: orotracheal intubation, OR: odds ratio, PACA: Provence-Alpes-Côte d’Azur, PDL: Pays de la Loire, Ref: reference class, 95% CI: 95% confidence interval

Reading notes:

A patient may have several comorbidities.
