# Supplementary material for: Factors associated with an unfavorable outcome according to age in patients with COVID-19 admitted to intensive care in mainland France during the first three periods of the pandemic: a nationwide cohort study
Source: Front Med (Lausanne). 2026 Apr 23;13:1816657. doi: 10.3389/fmed.2026.1816657 (PMC13149367; doi:10.3389/fmed.2026.1816657)
Supplement: Supplementary file 11 [file Supplementary_file_11.docx]

Additional File 11: Quality of the multivariate models on the risk of using invasive respiratory support

|  | **<45 years** | **45-64 years** | **≥65 years** |
| --- | --- | --- | --- |
| C-statistic (95% CI) | 0.89 (0.86 – 0.91) | 0.88 (0.87 – 0.89) | 0.83 (0.82 – 0.84) |
| Hosmer-Lemeshow test | 0.76 | 0.25 | <0.001 |

Abbreviations:

95% CI: 95% confidence interval
